# Biomimetic nanotherapy: core–shell structured nanocomplexes based on the neutrophil membrane for targeted therapy of lymphoma

**DOI:** 10.1186/s12951-021-00922-4

**Published:** 2021-06-13

**Authors:** Qiangqiang Zhao, Duanfeng Jiang, Xiaoying Sun, Qiuyu Mo, Shaobin Chen, Wansong Chen, Rong Gui, Xianjun Ma

**Affiliations:** 1 Department of Blood Transfusion, The Third Xiangya Hospital, Central South University, Changsha, 410013 People’s Republic of China; 2 Department of Hematology, The Qinghai Provincial People’s Hospital, Xining, 810007 People’s Republic of China; 3 Department of Hematology, The Third Xiangya Hospital, Central South University, Changsha, 410013 People’s Republic of China; 4 Nursing School, Soochow University, Suzhou, 215000 People’s Republic of China; 5 Department of Emergency, The Qinghai Provincial People’s Hospital, Xining, 810007 People’s Republic of China; 6 Department of Hematology, Affiliated Hospital of Guilin Medical University, Guilin, 541002 People’s Republic of China; 7 College of Chemistry and Chemical Engineering, Central South University, Changsha, 410083 People’s Republic of China; 8 Department of Blood Transfusion, Qilu Hospital of Shandong University, Jinan, 250012 People’s Republic of China

**Keywords:** Neutrophil membrane, Mesoporous silica nanoparticles, Doxorubicin, Shanzhiside methylester, Lymphoma

## Abstract

**Background:**

Non-Hodgkin’s lymphoma (NHL) is a malignant disease of lymphoid tissue. At present, chemotherapy is still the main method for the treatment of NHL. R-CHOP can significantly improve the survival rate of patients. Unfortunately, DOX is the main cytotoxic drug in R-CHOP and it can lead to adverse reactions. Therefore, it is particularly important to uncover new treatment options for NHL.

**Results:**

In this study, a novel anti-tumor nanoparticle complex Nm@MSNs-DOX/SM was designed and constructed in this study. Mesoporous silica nanoparticles (MSNs) loaded with Doxorubicin (DOX) and anti-inflammatory drugs Shanzhiside methylester (SM) were used as the core of nanoparticles. Neutrophil membrane (Nm) can be coated with multiple nanonuclei as a shell. DOX combined with SM can enhance the anti-tumor effect, and induce apoptosis of lymphoma cells and inhibit the expression of inflammatory factors related to tumorigenesis depending on the regulation of Bcl-2 family-mediated mitochondrial pathways, such as TNF-α and IL-1β. Consequently, the tumor microenvironment (TME) was reshaped, and the anti-tumor effect of DOX was amplified. Besides, Nm has good biocompatibility and can enhance the EPR effect of Nm@MSNs-DOX/SM and increase the effect of active targeting tumors.

**Conclusions:**

This suggests that the Nm-modified drug delivery system Nm@MSNs-DOX/SM is a promising targeted chemotherapy and anti-inflammatory therapy nanocomplex, and may be employed as a specific and efficient anti-Lymphoma therapy.

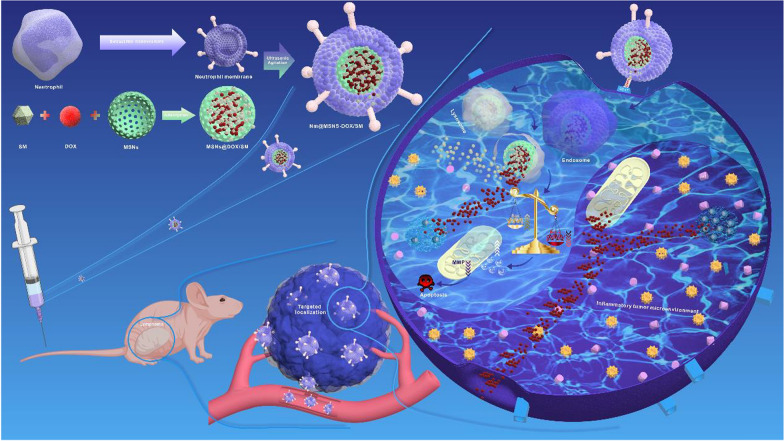

**Supplementary Information:**

The online version contains supplementary material available at 10.1186/s12951-021-00922-4.

## Background

Non-Hodgkin’s lymphoma (NHL) is a malignant disease of lymphoid tissue [1]. At present, chemotherapy is still the main method for the treatment of NHL [2]. Rituximab combined with cyclophosphamide, doxorubicin, vincristine, and prednisone (R-CHOP) can significantly improve the survival rate of patients [3]. Unfortunately, DOX is the main cytotoxic drug in R-CHOP. It can lead to adverse reactions such as myelosuppression and cardiotoxicity due to short half-life and non-specific distribution, limiting its clinical application [4]. Given these problems, nano-materials are loaded with chemotherapeutic drugs to improve the bioavailability of drugs and reduce the side effects of drugs [5]. Therefore, many different types of nanocarriers have been developed, such as inorganic mesoporous silica, quantum dots, metal nanoparticles, and liposomes [6].

With the development of nanotechnology, the nano-drug delivery system has been more and more widely used in tumor therapy, with great advantages [7, 8]. Silicon dioxide, which is broadly distributed in nature and has good compatibility, has been widely used in various cosmetics and some food additives [9]. Silica nanoparticles are one of the commonly used nanomaterials in industry, engineering, and biomedicine [10]. Among all the available nanomaterials, Mesoporous silica nanoparticles (MSNs) have attracted wide attention owing to good biocompatibility [11], high stability, adjustable pore structure, large loading, and easy surface functionalization [12, 13]. As an effective drug delivery system, MSNs have been used to treat various tumors [14, 15].

Inflammation plays a role in different stages of tumor development and has become the main marker of tumor [16]. In the inflammatory microenvironment of tumor, immune cells can release inflammatory factors such as TNF-α and IL-1β after immune cell infiltration [17], promote tumorigenesis, invasion and metastasis [18, 19]. Inflammation suppresses immune surveillance and immune regulation of the tumor, contributing to reducing the immune response of the host to the tumor [20]. Dead cells after DOX chemotherapy can stimulate immune cells to activate the inflammatory effect and further enhance tumor drug resistance, though nano-drugs can reduce the toxic and side effects caused by free drugs [21]. Since these factors can promote inflammation, failure of chemotherapy results in tumor recurrence [22]. Therefore, the combination of drugs and anti-inflammatory therapy is a promising anti-tumor strategy.

Shanzhiside methylester (SM) is a traditional Chinese medicine extracted from unique flavor containing iridoid glycosides [23]. Recent studies have revealed that SM attenuates cerebral ischemia–reperfusion injury in diabetic rats through an anti-inflammatory mechanism [24]. SM presents a significant anti-anxiety effect by regulating excitatory/inhibitory synaptic transmission and reducing the inflammatory response of basolateral amygdala [25]. Moreover, SM may block myocardial inflammatory cascade by inhibiting the NF-κB signal pathway, protecting myocardium from ischemic injury [26]. Therefore, SM may be an ideal natural compound to inhibit inflammation. However, no nano-carrier has been demonstrated to be loaded with both SM and DOX for anti-inflammatory and anti-tumor therapy.

Nano-drugs are easily scavenged by the reticuloendothelial system when entering the body as the exogenous substances [27]. However, an increasing number of studies found that biomimetic cell membrane-coated nano drug delivery systems can solve the mentioned problem. Hu et al. [28] developed a core–shell nanocarrier coated with platelet membrane for the targeted and site-specific delivery of extracellular active drugs and intracellular functional small molecule drugs, which could enhance antitumor efficacy. The research team developed a platelet membrane-coated biomimetic nanocarrier capable of sequentially targeting the bone microenvironment and myeloma cells, which could inhibit multiple myeloma growth and eradicate thrombotic complications [29]. Cai et al. [30] reassembled 89Zr-labeled cancer cell membranes into multicompartment membrane-derived liposomes for the PET-trackable tumor-targeted theranostics. Therefore, a growing number of researchers have improved the active targeting ability of the preparation by using biomimetic nanoparticles and other methods. Neutrophils are recognized as the first responders to tissue injury, and they play an essential role in host defense against infection. Besides, persistent neutrophil infiltration refers to a sign of chronic inflammation [31]. On the whole, the tumor microenvironment is characterized by chronic inflammation, and the tumor is considered a “wound that will not heal” [32]. Zhang et al. [33] found that PLGA nanoparticles camouflaged by neutrophil membranes can neutralize inflammatory mediators to treat chronic arthritis. Moreover, Chen et al. [34] used neutrophil membrane coated poly (latic-*co*-glycolic acid) nanoparticles to deliver carfilzomib to effectively treat metastatic cancer. Thus, neutrophil membrane-coated nanoparticles exhibit a strong potential to construct biomimetic nano drug delivery systems.

On this basis, Nm camouflage MSNs (Nm@MSNs) as drug carriers were constructed, loaded with DOX and SM as anti-tumor and anti-inflammatory drugs, respectively, for tumor synergistic therapy. Nm@MSNs can evade immune recognition, accumulate in the tumor site actively, and trigger the release of drugs in the tumor site with the help of the weakly acidic environment of the tumor. The results demonstrated that the combined application of DOX and SM inhibited the inflammation of tumor microenvironment and further enhanced the anti-tumor effect Thus, Nm@MSNs-DOX/SM nanocomplex has high drug loading, immune escape, anti-inflammatory, and anti-tumor activity, with a broad prospect of application.

## Materials and methods

### Materials

Mesoporous silica was purchased from nanoComposix (USA). Shanzhiside methylester was purchased from DESITE (China). DOX and Dialysis Membrane (2 kD) were purchased from Solarbio (China). Cy5, Hoechst 33342, and LysoTracker Green DND-26 were purchased from Yeasen Biotechnology (China). DSPE-PEG-FITC, MW:2000 purchased from Ruixi Biology Co., Ltd (China). Annexin V-FITC apoptosis detection kit was purchased from Beyotime Biotechnology (China). ROS assay kit was purchased from Beyotime Biotechnology (China). JC-1 MitoMP Detection Kit and cell counting kit-8 (CCK-8) were purchased from Dojindo Laboratories (Japan). Penicillin and streptomycin cocktail, fetal bovine serum (FBS), and RPMI-1640 were purchased from Life Technologies (USA). Anti-Bcl-2, anti-Bax, anti-TNF-α and anti-IL-1β, anti-CD47, and anti-SIRPα were manufactured by Cell Signaling Technology (USA). Anti-Ly6c was purchased from Abcam (UK). TNF-α ELISA. IL-1β ELISA was purchased from R&D Systems (China). HRP conjugated goat anti-rabbit IgG and HRP conjugated goat anti-mouse IgG were manufactured by Auragene Biotech (China). TdT in situ apoptotic kit was purchased from R&D Systems (China). Hematoxylin eosin (H&E) and DAPI were purchased from Servicebio Technology (China). Polycarbonate porous membrane syringe filters (200 nm) were provided by Whatman (USA).

### Cell culture and animal’s xenograft models

SU-DHL-2 cell was provided by the Cell Bank of the Chinese Academy of Sciences. All the cells were cultured in RPMI-1640 medium containing 10% fetal bovine serum and 1% penicillin–streptomycin at 37 °C and under 5% CO_2_. Six-week-old Balb/c-nude mice were purchased from Hunan SJA Laboratory Animal Co., Ltd (China). The lymphoma model was established by injecting 6 × 10^7^ cells SU-DHL-2 cells in 100 μl of the complete medium into the subcutaneous space of each mouse.

### Preparation of neutrophil membrane (Nm)

Nm was obtained as previously described with minor changes [33]. The whole blood of fresh mice was collected and anticoagulated with EDTA. Besides, 3% gelatin saline was mixed with the whole blood of the same volume, and the red blood cells were deposited for 30 min. The upper suspension was added to the centrifuge tube containing Ficoll-Paque PLUS lymphocyte separation solution and centrifuged for 20 min at 1200*g*. The supernatant was discarded. Then, it was washed twice with PBS (pH 7.4), centrifugated at 800*g* for 5 min to discard PBS. Afterward, 1 ml precooled double distilled water was added to resuscitate and divide neutrophils. Additionally, ultrasonic treatment was performed at 42 kHz for 5 min, and three freezing cycles were conducted (frozen at − 80 °C for 30 min, and thawed for 30 min at room temperature). It was centrifuged at 4 ℃ for 30 min (700 g × 10 min) to collect 14,000*g* supernatant. The Nm was obtained after discarding the supernatant and collecting precipitation.

### Preparation of MSNs-DOX/SM

MSNs (5 mg), DOX (2 μmol), and SM (40 μmol) were put into the total volume of 1 ml PBS, respectively. After stirring for 24 h at room temperature, the free DOX and SM were removed by dialysis. The remaining fluid after dialysis was used to determine the concentration of DOX and SM. Then, the encapsulation efficiency (EE) and drug load efficiency (LE) of two drugs were calculated with the previous method [35].

### Construction of Nm@MSNs-DOX/SM

To prepare Nm@MSNs biomimetic nanoparticles, neutrophil membranes were mixed with MSNs in PBS at a mass ratio of 1:1–8:1, polycarbonate membranes at 400 and 200 nm were sequentially extruded with a liposome extruder, and the unsuccessfully loaded neutrophil membranes were purified and then removed through the centrifugation (10,000×*g*, 10 min). In addition, the content of membrane proteins on Nm@MSNs was determined with a BCA kit [36] to determine the membrane-to-nucleus mass ratio with the highest neutrophil membrane coating efficiency. Nm vesicles were mixed with equal volume MSNs@DOX/SM and treated with ultrasound (2 min, 42 kHz, 80 W). Then, the mixture was filtered 10 times with a 200 nm porous membrane filter. Nm@MSNs-DOX/SM was obtained after the removal of free Nm by centrifugation (3000 rpm, 5 min, 4 ℃).

### Characterization of Nm@MSNs-DOX/SM

Particle size and morphology of MSNs, Nm, and Nm@MSNs were evaluated by Tecnai G2 Spirit TEM (FEI, USA) to confirm that the Nm wrapped on the surface of MSNs. The particle size distribution and Zeta potential were determined by Zetasizer Nano ZS (Malvern Nano series, Malvern, UK). Absorbances were determined using UV/vis spectrometry (scandrop, Analytik Jena, Germany). Nm proteins were identified by Silver staining [37]. Silver staining was performed with the Fast Silver Stain Kit (Beyotime, Haimen, China) as the protocol described while MS was completed by Wininnovate Bio (Shenzhen, China). Afterward, protein identification and quantification were accomplished by Proteome Discoverer software (version 1.4; Thermo Fisher Scientific, USA).

### DOX and SM release features of Nm@MSNs-DOX/SM

The drug release tests were performed under pH 7.4 and pH 5.0 to determine whether DOX and SM could be released in a pH-dependent manner. Dialysis was applied to analyze the release of DOX and SM from MSNs@DOX/SM and Nm@MSNs-DOX/SM. Dialysis bags (molecular weight cut-off, MWCO 8000–14,000 Da) were filled with 2 ml of MSNs@DOX/SM and Nm@MSNs-DOX/SM, respectively. The bags were immersed in 20 ml of PBS containing 0.2% (w/v) Tween 80 and stirred at 100 rpm and 37 °C [38, 39]. The absorbance values of SM and DOX in dialysate were determined at 238 nm and 480 nm, respectively. The cumulative release of SM and DOX were based on the standard curve.

### Biocompatibility of Nm@MSNs

The biocompatibility of Nm@MSNs was evaluated by hemolysis rate [40]. The Nm@MSNs samples of different concentrations were mixed with 5% nude mouse erythrocyte suspension, incubated at 37 °C for 2 h and centrifuged at 3500 rpm for 5 min. The supernatant was collected, and its absorbance was measured at 540 nm. Ultra-pure water and PBS were used as positive control and negative control. Then, the hemolysis rate was calculated. Besides, RAW264.7 cells were inoculated into a six-well plate at a density of about 1 × 10^5^ cells/well to determine the immune escape function of Nm@MSNs. Nm@MSNs-RhB and MSNs-RhB were added to RAW264.7 cells, respectively, incubated for 6 h [41]. The nucleus was stained with Hoechst 33342. The phagocytosis of macrophages to Nm@MSNs was observed under laser confocal microscope (LCFM, LSM700, Germany).

### Evaluation of in vitro anti-inflammatory effects of Nm@MSNs-DOX/SM

Inflammation can lead to an increase in the expression of pro-inflammatory cytokines, and macrophages can secrete pro-inflammatory cytokines [42]. The expression levels of IL-1β and TNF-α were detected by enzyme-linked immunosorbent assay (ELISA). RAW264.7 cells were seeded in a 6-well plate at a density of about 1 × 10^5^ cells/well and then added to each well for 12 h. PBS, MSNs, SM, MSNs@SM, and Nm@MSNs-SM were added to a 6-well plate for 24 h. Next, the supernatant was collected by centrifugation (1000 rpm, 5 min), and the contents of TNF-α and IL-1β in the supernatant were determined according to ELISA instructions. The percentage of cytokines was calculated based on the reported method [43].

### Evaluation of in vitro anti-tumor effects of Nm@MSNs-DOX/SM

SU-DHL-2 cells were inoculated on a 96-well plate, and the cell inoculation density was 2 × 10^3^/well. The cells were treated with PBS, SM, DOX, DOX + SM, MSNs@DOX/SM, and Nm@MSNs-DOX/SM groups, respectively. After cultured at 37 °C and 5% CO_2_ for 24 h, 10 μl CCK-8 was added to each pore and incubated in 37 °C and 5% CO_2_ for 4 h. Then, the absorbance at 450 nm was measured by a microplate reader (PerkinElmer EnSpire, USA). Afterward, cells were stained alive/dead with Calcein-AM and PI and then observed by inverted fluorescence microscope (ZEISS Axio Vert.A1, Germany).

### Effects of Nm@MSNs-DOX/SM on apoptosis, ROS, and MMP on SU-DHL-2 cells

SU-DHL-2 cells (5 × 10^5^ cells/well) were inoculated and treated with PBS, SM, DOX, DOX + SM, MSNs@DOX/SM, and Nm@MSNs-DOX/SM for 24 h, respectively. After 24 h, cells were collected, washed, and suspended with PBS. JC-1 Assay Kit and ROS Assay Kit were adopted to detect the MMP and ROS level of SU-DHL-2 cells, respectively, with flow cytometry. Annexin V-FITC Apoptosis Detection Kit was used to detect cell apoptosis with flow cytometry.

### Nm@MSNs distribution assay in vivo

Nm@MSNs labeled by Cy5 were compared with MSNs labeled by Cy5 to evaluate target of Nm@MSNs in vivo. Cy5-Nm@MSNs were injected into caudal vein at the dose of 1 µg/kg. The fluorescence intensity at 6, 24, and 48 h after administration was detected on the Xenogen IVIS Lumina XR imaging system (Caliper Life Sciences, USA) [44]. MSNs-Cy5 and Nm@MSNs-Cy5 were injected into the tail vein of nude mice 48 h later, respectively, and the main tissues and organs (i.e., heart, liver, spleen, lung, kidney and tumor) were removed, and the imaging was further verified thorough the fluorescence imaging. The results are presented as the percentage injected dose per gram of tissue (i.e., %ID/g) [45, 46].

### Nm@MSNs-DOX/SM treatment in lymphoma-bearing mice

When the tumor volume reached 100 mm^3^, the transplanted tumor nude mice were randomly divided into six groups with three mice in each group. The administration times of 100 ul PBS, SM, DOX, DOX + SM, MSNs@DOX/SM, and Nm@MSNs-DOX/SM in tail vein of nude mice were once every 2 days and 4 times in a row. The dosage of DOX and SM was 2 mg/kg [47] and 40 mg/kg [24], respectively. The tumor length (L, mm) and width (W, mm) were measured with an electronic vernier caliper, and the tumor volume (V, mm^3^) was calculated by the calculation formula V = (LW^2^)/2, which was monitored every 3 days to calculate the tumor volume size. Following the manufacturer’s instructions, the apoptotic nuclei were examined with TDT in situ apoptosis kit. Frozen tumor tissue into frozen section, the detection of MMP and ROS in vivo was based on the standard scheme of JC-1 and DCFH-DA immunofluorescence staining.

### Nm@MSNs-DOX/SM in vivo safety evaluation

Since the constructed Nm@MSNs-DOX/SM nanocomposite is an allogeneic substance, the verification of its safety is essential for its application in organisms. In this study, its safety was verified in terms of body weight, blood cell count, serum enzymology, and H&E staining of major tissues. The body weight of all nude mice was recorded per 3 days. At the end of the in vivo tumor treatment experiment, mouse blood was collected and anticoagulated with sodium citrate through the eyeball blood sampling, and it was allowed to stand for 2 h. Then, supernatant plasma was collected through the centrifugation at 3500 rmp. White blood cell (WBC), hemoglobin (HGB) and platelet (PLT) were detected by using the automatic blood cell analyzer (BC-5390, Mindray, Shenzhen, China). Automatic biochemical instrument (7100, HITACHI, Tokyo, Japan) was employed to detect alanine aminotransferase (ALT) and aspartate aminotransferase (AST), while renal function was measured by creatinine (CRE) and blood urea nitrogen (BUN) and cardiac function was measured with creatine kinase (CK) and myoglobin (Myo). Afterwards, the collected heart, liver, spleen, lung and kidney tissue sections were stained with H&E, first routinely deparaffinized to water, dripped with hematoxylin to be stained for 10 min, washed with water for 2 min, then differentiated with 1% hydrochloric acid in 75% alcohol for 3 s and then rinsed with tap water for 10 min. Water-soluble eosin staining was added dropwise for 2 min, and then the washing was conducted with distilled water for 60 s. Subsequently, the sections were deparaffinized with alcohol-dehydrated xylene, mounted with neutral gum for microscopic examination, and the tissue status was observed and photographed under a light microscope.

### Western blotting analysis and immunofluorescence staining

The total protein was extracted from cell lysate with RIPA buffer. The total protein concentration was determined by BCA protein detection kit. The protein expression levels of Bcl-2, Bax, CD47, anti-SIRPα, TNF-α, IL-1β and Ly6c were detected by the WB method. Paraffin-embedded tissue samples were prepared for detachment and antigen retrieval. According to the standard scheme, anti-Bcl-2, anti-Bax, anti-TNF-α, and anti-IL-1β were used for immunofluorescence staining. Subsequently, the nucleus was stained with DAPI. Finally, images were observed and taken under a fluorescence microscope.

### Statistical analysis

SPSS 22.0 software was used for statistical analysis. Data were expressed as the mean ± SD. Differences between groups were assessed by one-way ANOVA, followed by Tukey’s post-test (*p < 0.05, ** *p* < 0.01, and *** *p* < 0.001).

## Result

### Preparation and characterization of Nm@MSNs-DOX/SM

**Fig. 1:**
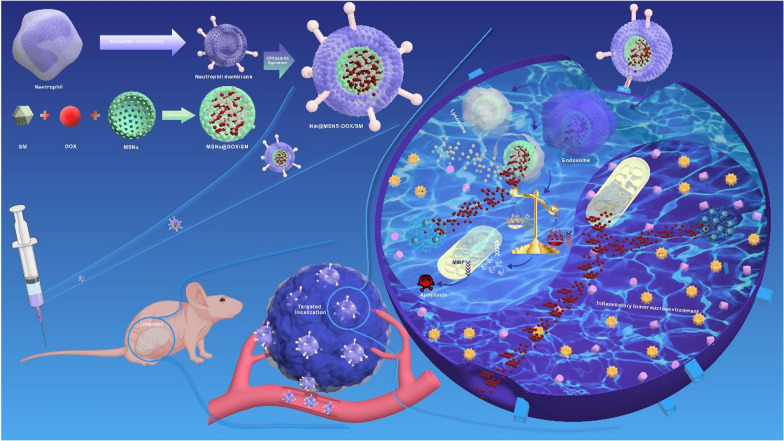
Schematic diagram of Nm@MSNs-DOX/SM construction and its targeted therapeutic mechanisms in lymphoma

**Fig. 2:**
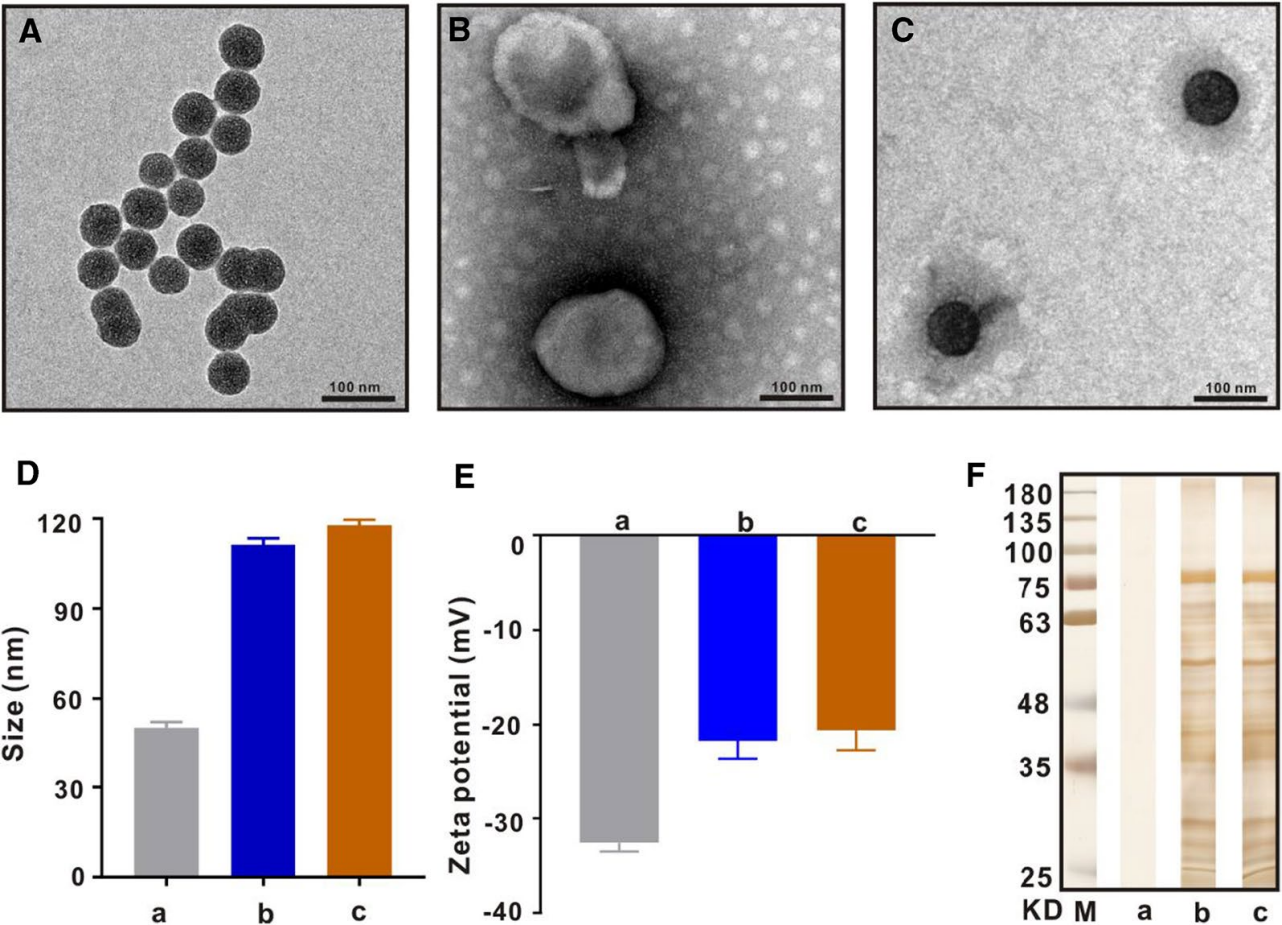
Characteristics of Nm@MSNs-DOX/SM. TEM images: **A** MSNs, **B** Nm vesicles, **C** Nm vesicles camouflage MSNs. Scale bar: 100 nm. **D**, **E** The particle size and Zeta potential of (a) MSNs, (b) Nm vesicles and (c) Nm vesicles camouflaged MSNs, respectively. **F** Silver staining analysis. (M) markers, (a) MSNs, (b) Nm vesicles, and (c) Nm vesicles camouflage MSNs. Data are mean ± SD (n = 3)

As illustrated in Fig. 1, the preparation process of Nm@MSNs-DOX/SM mainly consists of two steps: (1) DOX and SM were loaded into MSNs to obtain MSNs@DOX/SM; (2) Nm vesicles were wrapped in MSNs@DOX/SM through ultrasound. As revealed from the TEM image (Fig. 2A), the silica nanospheres are monodispersed with a diameter of about 50 nm. After 1% phosphotungstic acid negative staining, TEM presented that the Nm successfully wrapped MSN, with a spherical core–shell structure (Fig. 2B, C). According to the dynamic light scattering (DLS) analysis (Fig. 2D), the average particle size of Nm@MSNs nanocomposites is 121.6 ± 3.8 nm (close to 119.3 ± 4.2 nm of Nm). After MSNs were coated with Nm, its nanoparticle size increased from 50 to 120 nm. Besides, since the average thickness of cell bilayers was 7–12 nm, 70 nm of increased nanoparticle size might be caused by membrane multilayer coatings [48–50]. The Zeta potential of MSNs was − 32.6 ± 0.9 mV while the Zeta potential of Nm@MSNs nanocomposites was − 20.3 ± 2.1 mV, which was close to the Zeta potential of Nm vesicles (− 21.7 ± 1.8 mV) (Fig. 2E). Silver staining results (Fig. 2F) suggest that most of the Nm proteins encapsulate MSNs and eventually form Nm@MSNs nano-biomimetic materials. According to Additional file 1: Figure S1A, a good correlation was identified between the membrane protein content on the surface of biomimetic nanoparticles and the mass ratio of Nm-MSNs below 6:1, whereas the membrane protein content on the surface of biomimetic nanoparticles began to saturate when reaching over 6:1, which demonstrated that the neutrophil membrane could completely encapsulate MSNs at the mass ratio of 6:1. According to the UV–vis spectra (Additional file 1: Figure S1B), the absorption peaks of Nm@MSNs-DOX/SM are 295.5 nm, 203 nm, 480 nm, and 238 nm, respectively, consistent with the absorption peaks of Nm, MSNs, DOX and SM detected alone. These results confirm that Nm@MSNs-DOX/SM has been successfully synthesized.

### Drug loading rate and release rate

**Fig. 3:**
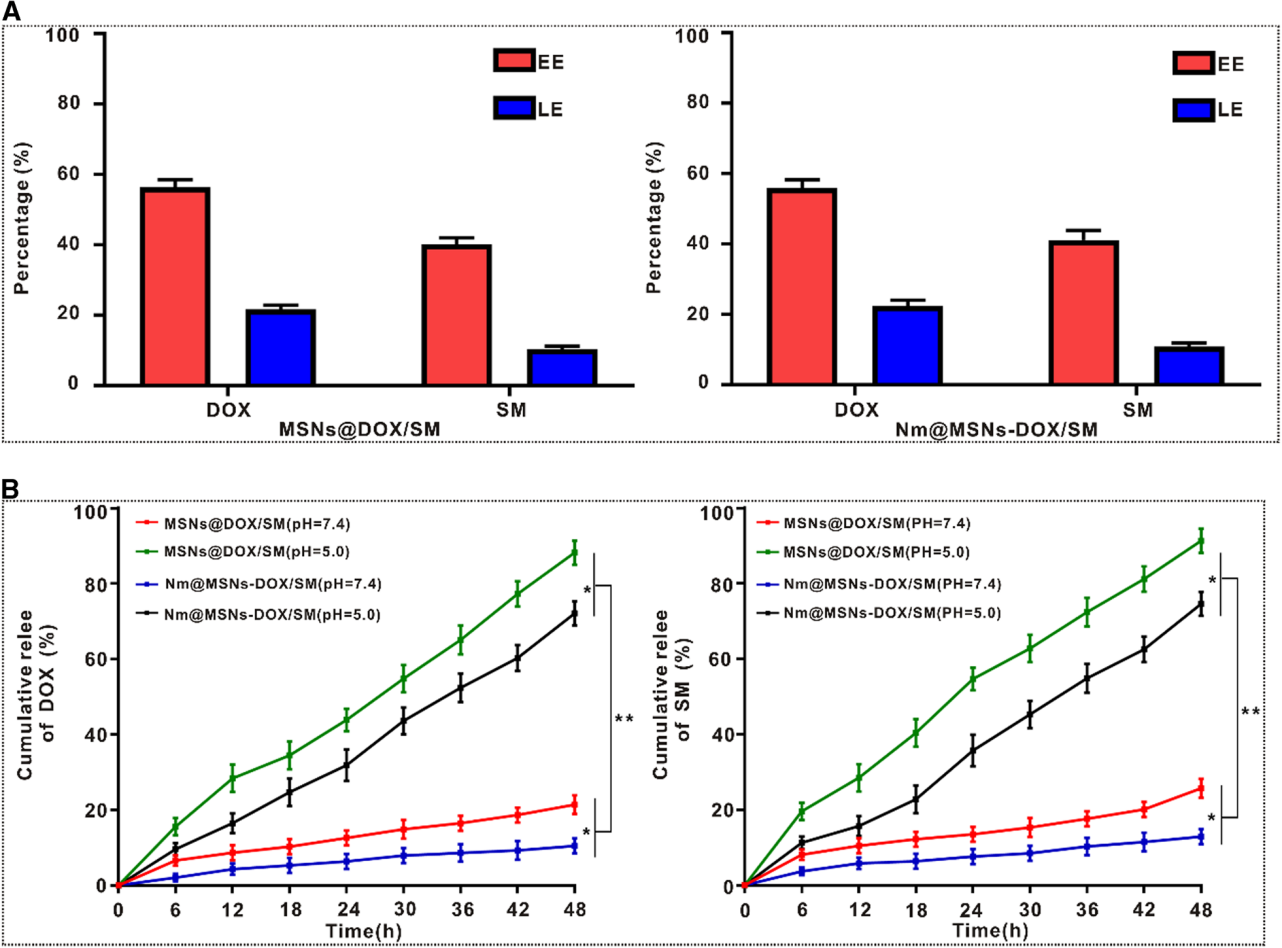
Drug loading and release. **A** EE and LE of MSNs and Nm@MSNs, respectively. **B** The cumulative release rate of DOX and SM from MSNs@DOX/SM or Nm@MSNs-DOX/SM, respectively, at different pH values (7.4 and 5.0). Data are mean ± SD (n = 3), (*p < 0.05 and **p < 0.01)

Considering that MSNs has high specific surface area and good biocompatibility, it is an ideal carrier and has been used to load antineoplastic drugs in many studies. According to Fig. 3A, DOX and SM were packaged into MSNs with drug EE (DOX 56.4 ± 2.14%, SM 40.2 ± 1.83%) and LE (DOX 21.7 ± 1.26%, SM 10.4 ± 0.82%), respectively, while drug EE (DOX 55.9 ± 2.69%, SM 41.1 ± 2.85%) and LE (DOX 22.3 ± 1.78%, SM 10.8 ± 1.13%) were packaged into Nm@MSNs. Besides, the drug release rates of MSNs@DOX/SM and Nm@MSNs-DOX/SM were measured. As exhibited in Fig. 3B, at pH7.4, DOX and SM released from Nm@MSNs-DOX/SM and MSNs@DOX/SM were 10.5% and 21.4%, 12.9% and 25.7%, respectively, from the beginning of the release to the end of 48 h of detection. At pH5.0, DOX and SM released by Nm@MSNs-DOX/SM and MSNs@DOX/SM within 48 h were 72.1% and 88.2%, 74.6% and 91.3%, respectively. In conclusion, the cumulative release rate of Nm@MSNs-DOX/SM and MSNs@DOX/SM under pH5.0 was significantly higher than that under pH7.4. The pH of normal human tissues and blood is around 7.4, while the extracellular microenvironment of tumor tissues presents a slightly acidic (pH 6.8–7.2) [51]. In tumor cells, the pH of lysosomes and endosomes decreased (pH 4.0–6.0) [52], so when Nm@MSNs-DOX/SM entered the lysosomes, the change of pH gradient between the normal tissue and the lysosome was exploited to facilitate the drug release in tumor. Meanwhile, the cumulative release rate of DOX and SM from Nm@MSNs was lower than that from MSNs, indicating that Nm inhibited the rapid release of drugs from MSNs to some extent and played the role of sustained-release drugs. To sum up, Nm@MSNs are an efficient drug carrier in vitro, and acidic environment can promote the release of DOX and SM from Nm@MSNs.

### Biocompatibility and immune escape of Nm@MSNs-DOX/SM

**Fig. 4:**
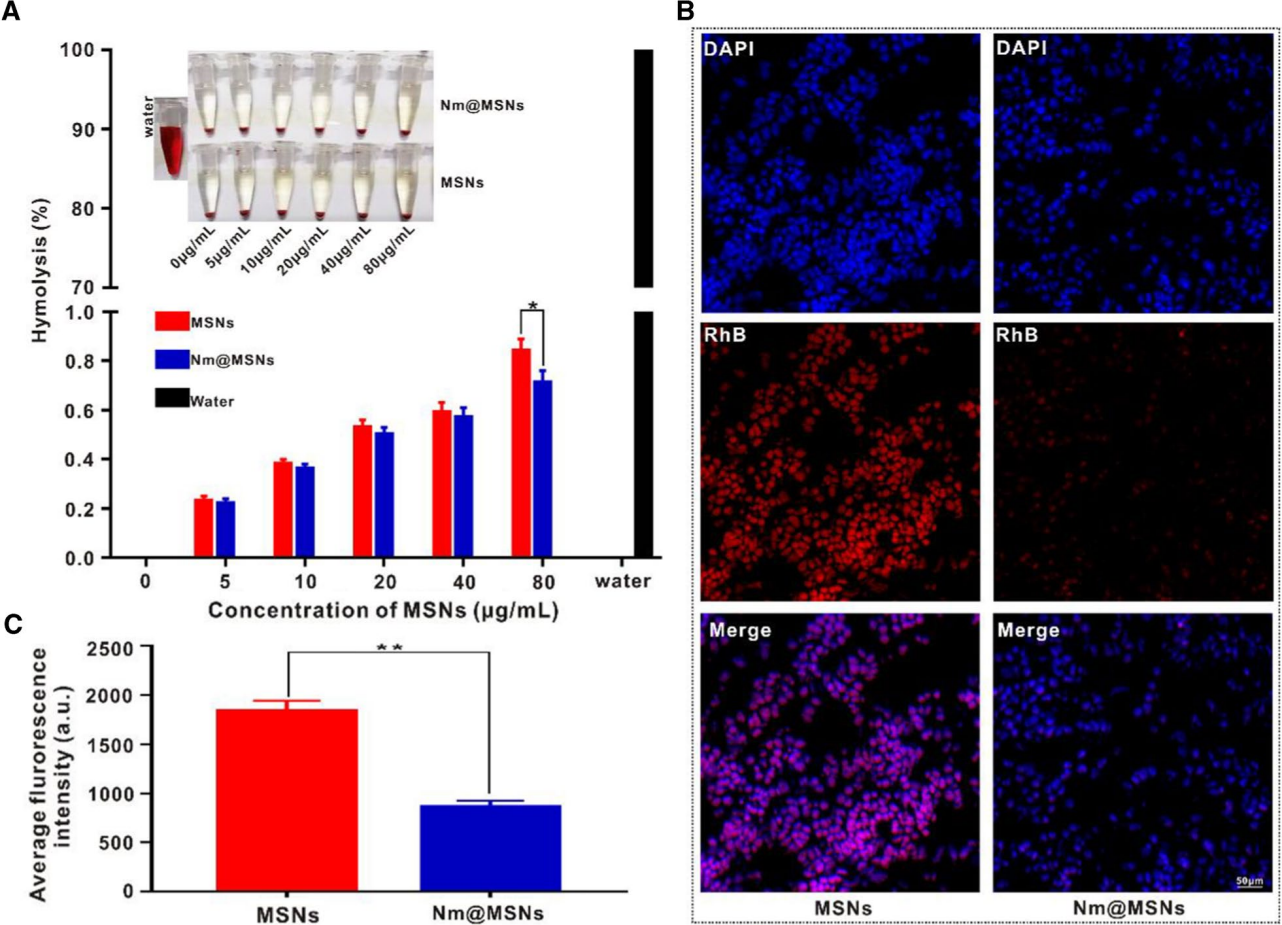
Biocompatibility of Nm@MSNs. **A** Hemolysis rate of red blood cells in different concentrations of MSNs and Nm@MSNs, respectively. **B** LCFM images of RAW264.7 macrophages co-cultured with MSNs-RhB and Nm@MSNs-RhB for 6 h, respectively. Scale bar: 50 μm. **C** Fluorescence intensities of collected cells after treatment with MSNs-RhB and Nm@MSNs-RhB, as quantified by a fluorometre. Data are mean ± SD (n = 3), (*p < 0.05 and **p < 0.01)

The biocompatibility of Nm@MSNs-DOX/SM was evaluated by hemolysis rate. In Fig. 4A, the concentration gradients of MSNs and Nm@MSNs-DOX/SM were constructed, and these nanocomplexes were added to erythrocytes, respectively, without significant hemolysis after 2 h (< 1%). However, Nm@MSNs lead to lower hemolysis compared to MSNs, suggesting that Nm@MSNs are better than MSNs in biosafety. Next, the cell viability of SU-DHL-2 cells was determined by CCK-8. The cell survival rate of SU-DHL-2 cells incubated with a series of concentrations of MSNs or Nm@MSNs for 24 h, of which the survival rate of SU-DHL-2 cells treated with 80 μg/ml NM@MSNs, was observed to be as high as 90% (Additional file 1: Figure S2). MSNs and Nm@MSNs were labeled with RhB to detect the anti-phagocytosis effect. As observed from Fig. 4B, bright red fluorescence appeared 6 h after MSNs-RhB was added to RAW264.7 cells, demonstrating that macrophages swallowed up a considerable amount of MSNs-RhB. However, weak fluorescence of Nm@MSNs-RhB was observed under the same conditions, reflecting that the phagocytosis of RAW264.7 cells was effectively inhibited. The average fluorescence intensity of macrophages treated with Nm@MSNs-RhB was significantly lower than that treated with MSNs-RhB (Fig. 4C). These results verify that MSNs camouflaged by Nm can escape the recognition of macrophages and reduce the phagocytosis and clearance rate of macrophages. Thus, Nm@MSNs have the ability of immune escape.

### Anti-inflammatory effect of Nm@MSNs-SM in vitro

The inflammatory microenvironment around tumor plays an essential role in tumor formation, growth, and metastasis [53]. In this study, the role of SM is to use its anti-inflammatory effect on the surrounding area of the tumor to achieve the effect of combined therapy. Macrophages were stimulated by lipopolysaccharide (LPS) to induce inflammation, and the anti-inflammatory effects of SM, MSNs@SM, and Nm@MSNs-SM were evaluated (the concentration of SM in the above-mentioned groups was identical). As presented in Additional file 1: Figure S3, the levels of TNF-α and IL-1β in the supernatant were significantly increased by ELISA assay after RAW264.7 cells were stimulated by LPS, demonstrating that the inflammatory model in vitro was successfully established. SM, as a bioactive and anti-inflammatory component, can reduce the secretion of TNF-α and IL-1β by 31.6% and 25.2%, respectively. However, the ELISA and Western blotting (Additional file 1: Figure S4A–C) results shown that the anti-inflammatory activity of Nm@MSNs-SM is higher than that of SM because Nm prevents the phagocytosis of phagocytes. In short, Nm@MSNs-SM can play its anti-inflammatory effect by releasing SM.

### Targeting and antigen functionalization of neutrophil membrane in vitro

**Fig. 5:**
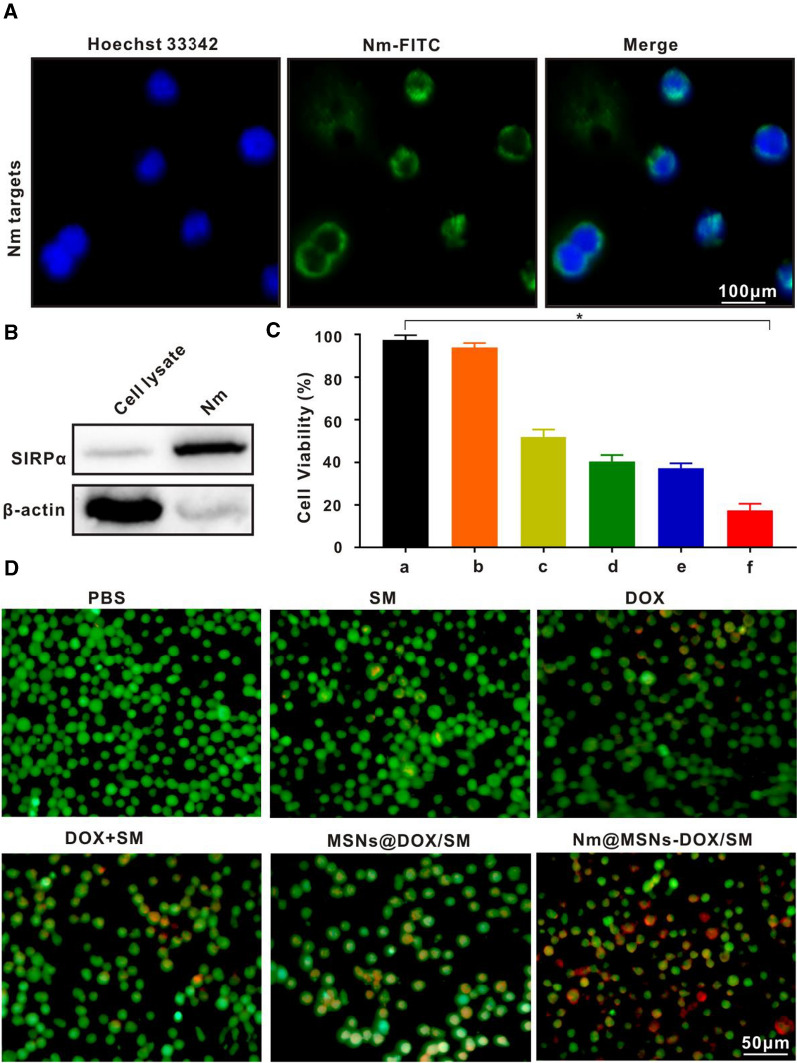
Verify the targeting of Nm and the anti-tumor effect of Nm@MSNs-DOX + SM in vitro. **A** The LCFM images of DSPE-FITC-labeled Nm vesicles were co-cultured with SU-HDL-2 cells for 6 h. Scale bar: 100 μm. **B** Detection of membrane specific protein SIRPα by Western blotting. **C** Survival rate of SU-HDL-2 cells treated with (a) PBS, (b) SM, (c) DOX, (d) DOX + SM, (e) MSNs@DOX/SM and (f) Nm@MSNs-DOX/SM for 24 h, respectively. **D** Live/dead staining of SU-HDL-2 cells upon various treatments for 24 h. Scale bar: 50 μm. Data are presented as the mean ± SD (n = 3). (*p < 0.05)

CD47 is a molecular marker expressing “self-recognition” on the cell surface [54], which is significantly overexpressed in various kinds of B-cell lymphoma [55]. CD47 sends immunosuppressive signals downstream by binding to the receptor (signal regulatory protein α, SIRPα) expressed on the surface of neutrophils [56, 57]. Ly6c is a 14kd protein found to be highly expressed in neutrophils [58], monocytes [59] and dendritic cells [60], in which neutrophils is the most prominent [61]. Besides, it is a neutrophil-specific membrane protein that plays a crucial role in neutrophil activation, migration and chemotaxis [62, 63]. Additional file 1: Figure S5A-B illustrates that CD47 was highly expressed in SU-DHL-2 cells. Next, the targeting of Nm vesicles to lymphoma cells was verified. In Fig. 5A, green fluorescent Nm vesicles gather around the nucleus of SU-DHL-2, suggesting that Nm vesicles may adhere to SU-DHL-2 cells and have active targeting. In Fig. 5B and Additional file 1: Figure S5C–D, the specific neutrophil membrane marker SIRPα and Ly6c were highly expressed. Thus, β-actin, as a marker of cytoplasmic protein, mainly existed in the bands of cell lysate.

### Antitumor effect of Nm@MSNs-DOX/SM in vitro

Considering that inflammation plays a crucial role in the formation of tumor, we assume that chemotherapy combined with anti-inflammatory therapy can improve the effect of anti-tumor. The antitumor activity of Nm@MSNs-DOX/SM, MSNs@DOX/SM, DOX + SM, DOX, SM, and PBS, in vitro was evaluated by cytotoxicity, Live/dead staining, alteration of reactive oxygen species (ROS), mitochondrial membrane potential (MMP), and cell apoptosis, respectively. To investigate the antitumor effects of DOX and SM, the two drugs were incubated with SU-DHL-2 cells for 24 h, respectively, and their cytotoxicity was detected by using CCK-8. According to Additional file 1: Figure S6A–B, the inhibitory effect of DOX was strong and exhibited a significant dose response, with an IC50 of approximately 2.0 μmol/l, SM hardly affected SU-DHL-2 cell viability. The combination index (CI) of DOX and SM combination was calculated by using compusyn software [64], and at the molar ratio of DOX and SM reaching 1:20, the best synergistic action was achieved. Thus, the DOX and SM were used at 1:20 (i.e., DOX 2 μmol/l, SM 40 μmol/l) to construct the novel anti-tumor nanoparticle. To compare the antitumor effects of Nm@MSNs-DOX/SM, MSNs@DOX/SM, DOX + SM, DOX and SM, the same concentration of DOX + SM was used (i.e., DOX 2 μmol/l, SM 40 μmol/l). It is DOX + SM (i.e., DOX 2 μmol/l, SM 40 μmol/l) in these two groups of Nm@MSNs-DOX/SM and MSNs@DOX/SM for cell viability studies. The results of CCK-8 (Fig. 5C) revealed that the survival rate of SU-DHL-2 cells treated with free drug DOX decreased by 50.8 ± 4.2% compared with the control group PBS. However, the rate of killing SU-DHL-2 cells reached 61.4 ± 3.6% when SU-DHL-2 cells were treated with DOX combined with SM. Nm@MSNs-DOX/SM had a significant killing effect on SU-DHL-2 cells (83.5 ± 5.2%). The SU-DHL-2 cells were treated with Nm@MSNs-DOX/SM, MSNs@DOX/SM, DOX + SM, DOX, SM, and PBS for 24 h, respectively. Propidium iodide (PI) staining showed red fluorescence. Specifically, the stronger the red fluorescence, the more the dead cells. The staining images of living/dead cells were also consistent with the results of CCK-8 (Fig. 5D). The results further confirmed that Nm@MSNs-DOX/SM had good anti-tumor effect in vitro.

### Effects of Nm@MSNs-DOX/SM on ROS, apoptosis, MMP and apoptosis proteins on SU-DHL-2 cells

**Fig. 6:**
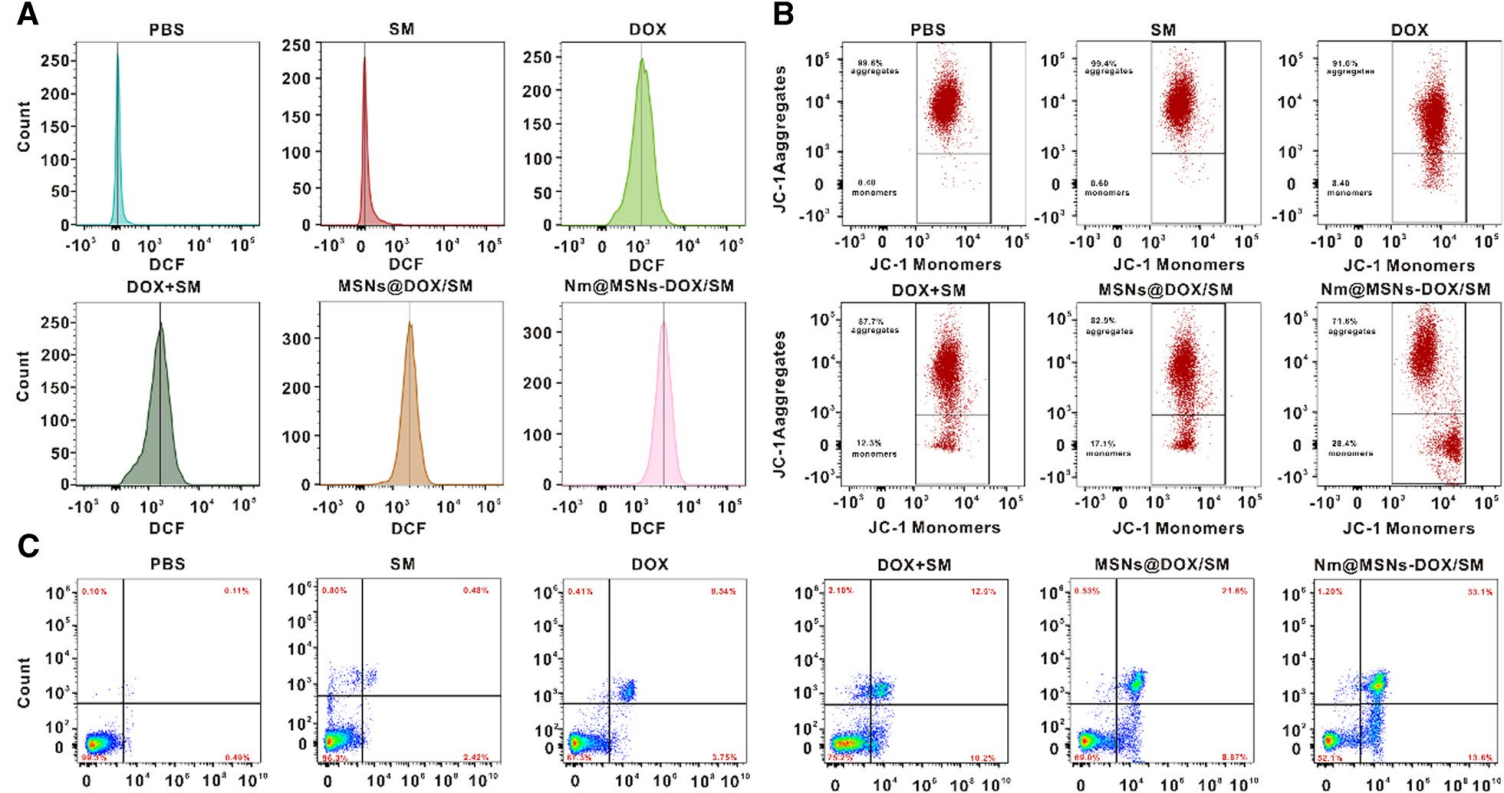
Anti-lymphoma effect of Nm@MSNs-DOX/SM in vitro was detected by flow cytometry. **A** ROS level of SU-HDL-2 cells was detected by flow cytometry after 24 h. **B** Flow cytometry was used to detect the changes of MMP in SU-HDL-2 cells after 24 h. **C** Flow cytometry was used to detect the apoptosis of SU-HDL-2 cells after 24 h

ROS, MMP, apoptosis, and apoptosis-related genes were detected to further explore the anti-tumor mechanism of Nm@MSNs-DOX/SM in vitro. The physiological level of ROS can mediate the normal physiological function of cells while excessive ROS can lead to cell injury and death [65]. ROS is an indicator of mitochondrial dysfunction, and its increase is a landmark event of early apoptosis [66]. The ROS levels of SU-DHL-2 cells in PBS, SM, DOX, DOX + SM, MSNs@DOX/SM, and Nm@MSNs-DOX/SM were detected by flow cytometry. As observed in Fig. 6A and Additional file 1: Figure S7A, the Nm@MSNs-DOX/SM caused the largest increase in the level of ROS compared with the other groups, demonstrating the strong anti-tumor effect of Nm@MSNs-DOX/SM nanocomplex in vitro. Furthermore, the effects of Nm@MSNs-DOX/SM on MMP of SU-DHL-2 cells by flow cytometry with JC-1 staining were evaluated. In Fig. 6B and Additional file 1: Figure S7B, the percentage of reduction to MMP reached 30.6 ± 3.1% after treatment with Nm@MSNs-DOX/SM, which was higher compared with other groups. Thus, Nm@MSNs-DOX/SM was inferred to damage the mitochondria of cells, leading to a decrease in MMP. The apoptosis of SU-DHL-2 cells was analyzed by flow cytometry (Fig. 6C and Additional file 1: Figure S7C). The results indicated that the apoptosis rate of SU-DHL-2 cells induced by DOX was 12.3 ± 1.1%, the apoptosis rate of SU-DHL-2 cells induced by DOX + SM group was 22.7 ± 1.8%, and the apoptosis rate of SU-DHL-2 cells treated by MSNs@DOX/SM group was 30.5 ± 2.6%. The percentage of SU-DHL-2 cell apoptosis induced by Nm@MSNs-DOX/SM was 46.7 ± 3.2%. As illustrated in Additional file 1: Figure S8A–C, free Dox can increase the expression of TNF-α and IL-1β protein while DOX + SM and Nm@MSNs-DOX/SM can lead to a decrease in the expression of these two proteins and a significant decrease in the expression of TNF-α and IL-1β protein, respectively. It was further verified that the nanocomplex can inhibit the expression of inflammatory factors. Therefore, Nm@MSNs-DOX/SM nanocomposite has both chemotherapy and anti-inflammatory properties, and better anti-tumor effect than DOX alone. Particularly, the endogenous pathway of mitochondria is the main pathway resulting in apoptosis [67]. Under normal circumstances, apoptosis is strictly regulated once the disorder can lead to tumors and other diseases [68]. The mitochondrial-mediated endogenous pathway regulated by the Bcl-2 family is considered the “tipping point” of apoptosis [69]. Bcl-2 protein plays an essential role in the regulation of apoptosis (widely expressed in most NHL [11]). It is mostly located in the mitochondrial intima of cells and inhibits apoptosis by regulating the expression of Bax protein [35]. As presented in Additional file 1: Figure S9, Nm@MSNs-DOX/SM can significantly inhibit the expression of Bcl-2 protein and then promote the expression of Bax protein, contributing to inhibiting the apoptosis of SU-DHL-2 cells. To sum up, Nm@MSNs-DOX/SM can inhibit SU-DHL-2 cells through anti-inflammation and inhibition of Bcl-2 family-mediated mitochondrial apoptosis pathway.

### Biodistribution of Nm@MSNs-DOX/SM in vivo

**Fig. 7:**
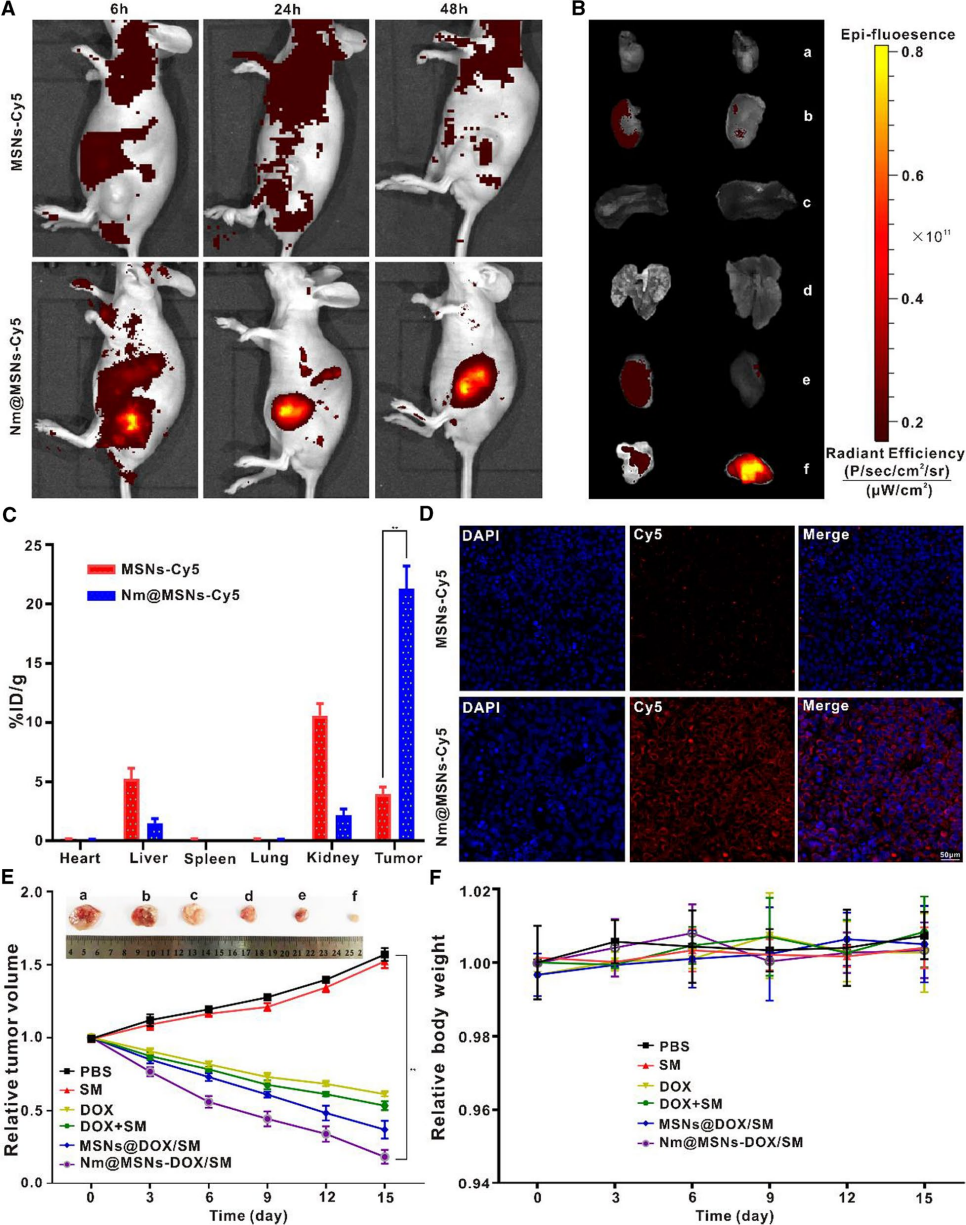
Verification targeting and distribution of Nm@MSNs-DOX/SMin vivo. **A** In vivo fluorescence images of SU-HDL-2 xenograft model at 6 h, 24 h and 48 h after intravenous injection of cy5-labeled Nm@MSN sand MSNs, respectively. **B** Ex vivo bioluminescent images of the main organs and tumours at 48 h post injection. (a) heart, (b) liver, (c) spleen, (d) lung, (e) kidney and (f) tumour. **C** The results are presented as the percentage injected dose per gram of tissue (i.e., %ID/g). **D** Fluorescence micrographs of tumour sections. Scale bar: 50 μm. **E** Curve changes of tumor volume in SU-DHL-2 tumor-bearing mice during various treatments for 15 days. The tumor tissues were treated with (a) PBS, (b) SM, (c) DOX, (d) DOX + SM, (e) MSNs@DOX/SM and (f) Nm@MSNs-DOX/SM, respectively. **F** The change curve of body weight of SU-DHL-2 tumor-bearing mice during treatment. Data are presented as the mean ± SD (n = 3), (*p < 0.05 and **p < 0.01)

As an effective anticancer drug delivery system, the ideal nanocarrier should be able to transfer the drug directly to the tumor tissue, so as to achieve tumor-targeted therapy. With the purpose of confirming our hypothesis, Nm@MSNs were labeled with fluorescent dye Cy5, and its biological distribution was detected by in vivo imaging technique. Simultaneously, Cy5 marker MSNs was used as a control. The results of Fig. 7A suggested that the fluorescence intensity of tumor site after injection of Nm@MSNs-Cy5 was higher than that of injection of MSNs-Cy5, demonstrating that Nm@MSNs had more accumulation in tumor site compared to MSNs. As shown in Fig. 7B, C that the uptake of NmMSNs-Cy5 group was 21.3 ± 1.9% ID/g, significantly higher than that of MSNs-Cy5 group. Additionally, the bright red fluorescence (DOX red fluorescence) in the tumor tissue of mice treated with Nm@MSNs-DOX/SM nanocomplex was more significant than that of mice treated with MSNs-DOX/SM (Fig. 7D). Thus, Nm@MSNS-DOX/SM has good active targeting and can increase the accumulation rate of drugs in tumors.

### Anti-inflammatory effect of Nm@MSNS-DOX/SM in vivo

The anti-inflammatory activity of Nm@MSNS-DOX/SM nanocomplex in vivo was detected by immunofluorescence staining to detect the TNF-α and IL-1β. As indicated in Additional file 1: Figure S10, the fluorescence signal of TNF-α and IL-1β in DOX was most evident, consistent with previous studies that DOX chemotherapy can increase the production of TNF-α and IL-1β and promote the proliferation and metastasis of tumor cells. However, the fluorescence signal of TNF-α and IL-1β decreased after combined with SM. Nm@MSNs-DOX/SM led to the most significant decrease of TNF-α and IL-1β fluorescence signal. These results suggest that DOX combined with SM can inhibit the expression of tumor-related inflammatory cytokines TNF-α and IL-1β, causing changes in the inflammatory microenvironment of tumor. It is further confirmed that Nm@MSNs-DOX/SM can inhibit the inflammation induced by LPS in vitro and tumor-related inflammation in vivo. Therefore, the synergistic effect of Nm@MSNs-DOX/SM in tumor therapy may be associated with the activation of anti-inflammatory effect.

### Antitumor effect of Nm@MSNs-DOX/SM in vivo

**Fig. 8:**
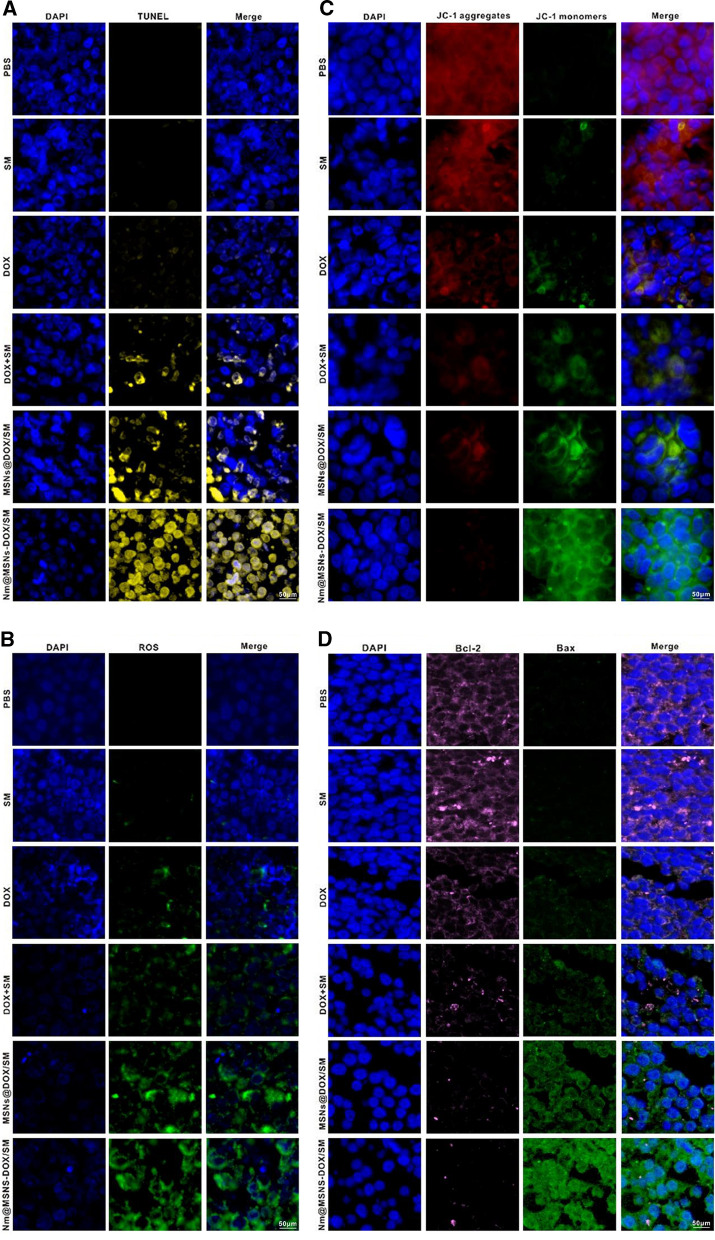
Anti-tumour effect of Nm@MSNs-DOX/SM in vivo. **A** After the tumor tissues were treated with different treatments for 15 days, TUNEL(yellow) was detected by immunofluorescence. **B**, **C** Tumor tissues were treated with different treatments, and immunofluorescence staining was used for ROS and MMP analysis. **D** The LCFM images of the tumor area were taken after different treatments. Pink: Bcl-2, green: Bax, blue: nucleus. Scale bar: 50 μm

Nm@MSNs-DOX/SM nanocomposites have an excellent anti-tumor effect in vitro. In this study, an in vivo model of SU-DHL-2 transplanted tumor in nude mice was investigated. As illustrated in Fig. 7E, after 15 days of treatment with DOX alone, the tumor tissue was significantly smaller than that treated with PBS alone. Compared with the free DOX, DOX combined with SM can further shrink the tumor tissue, confirming that DOX combined with SM can play a coordinating role and amplify the anti-tumor effect. However, Nm@MSNs-DOX/SM showed the strongest anti-tumor effect, significantly inhibiting the growth of tumor tissue. This can be explained by the possibility that free drugs entering the blood circulation may be cleared and swallowed by phagocytes while the camouflage of Nm plays a protective role. The role of Nm@MSNS-DOX/SM in inducing apoptosis in vivo is illustrated in Fig. 8A. TUNEL detection demonstrated that Nm@MSNS-DOX/SM could cause the strongest fluorescence signal (yellow) in tumor tissue, indicating that the percentage of apoptotic cells induced by Nm@MSNs-DOX/SM was significantly higher compared to other groups. After the tumors were treated with PBS, SM, DOX, DOX + SM, MSNs@DOX/SM, and Nm@MSNs-DOX/SM for 15 days, the green fluorescence signal in tumor tissue sections of Nm@MSNs-DOX/SM was the most significant, reflecting that Nm@MSNs-DOX/SM produced more ROS than DOX alone (Fig. 8B). Then, a small amount of red fluorescence and diffuse green fluorescence were observed in the tumor tissue sections of Nm@MSNs-DOX/SM. Therefore, the decrease of MMP induced by Nm@MSNs-DOX/SM was the most significant compared with other groups (Fig. 8C). The expression of Bcl-2 and Bax regulating apoptosis were detected by immunofluorescence. The pink (Bcl-2) fluorescence and green fluorescence (Bax) of tumor tissue sections treated with Nm@MSNs-DOX/SM are illustrated in Fig. 8D. The pink fluorescence was the weakest, and the green fluorescence was the strongest; the pink fluorescence gradually weakened, and the green fluorescence gradually increased in other groups. These results revealed that Nm@MSNs-DOX/SM treated tumor tissue inhibited the expression of Bcl-2 and promoted the expression of Bax.

### Evaluation of the safety of Nm@MSNs-DOX/SM in vivo

**Fig. 9:**
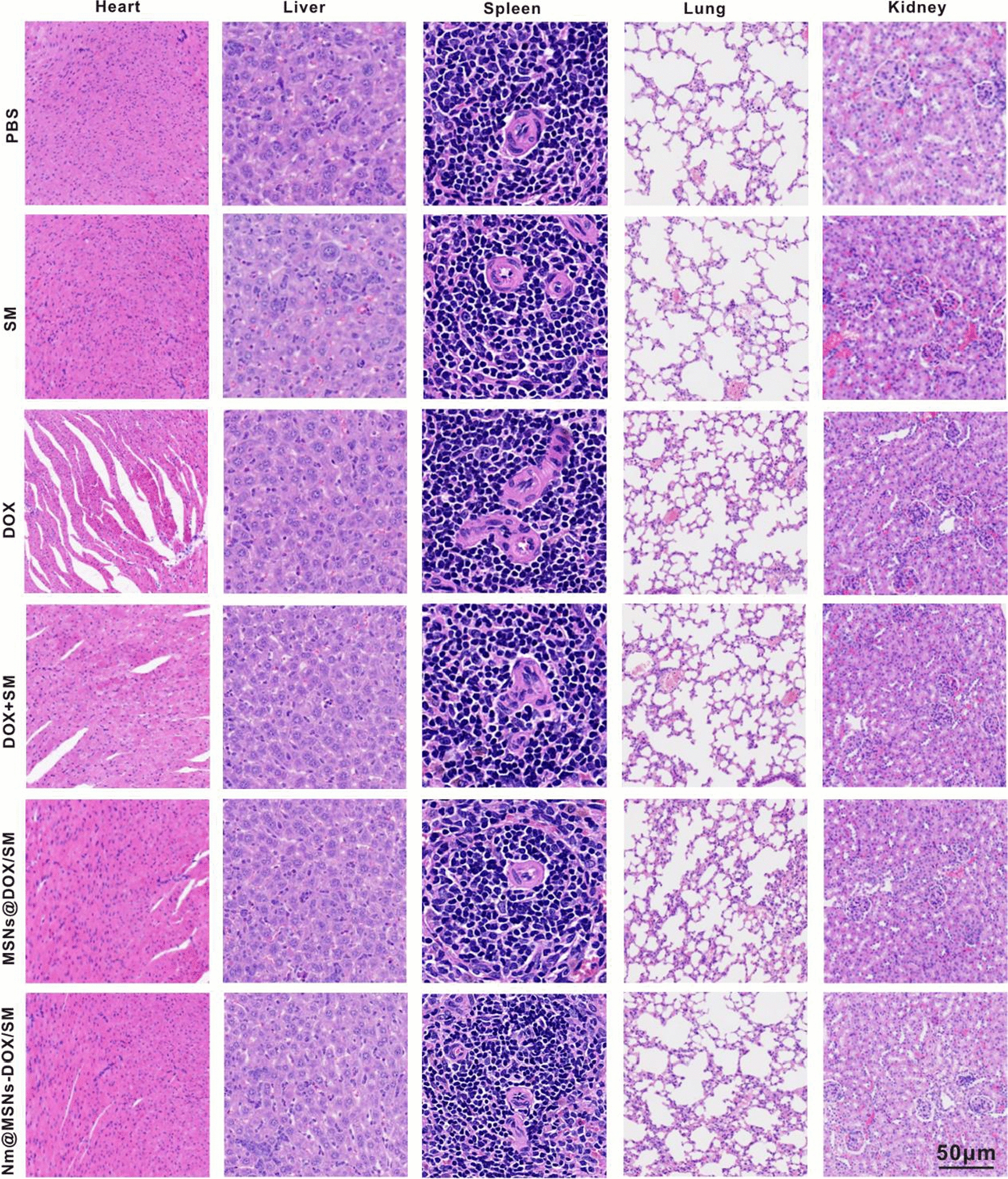
In vivo biological safety study. Nude mice were treated with PBS, SM, DOX, DOX + SM, MSNs@DOX/SM and Nm@MSNs-DOX/SM for 15 days, and the main organs were stained with H&E. Scale bar: 50 μm

The body weight of nude mice for 15 days was monitored to understand the potential toxicity of Nm@MSNs-DOX/SM. During the entire course of treatment, there were no dramatic changes in body weight in all the mice groups (Fig. 7F). As illustrated in Additional file 1: Figure S11, after treatment in DOX group and DOX + SM group, WBC and HGB count were all lower than other groups, suggesting that free drugs caused myelosuppression after chemotherapy. However, the WBC and HGB counts of the nude mice treated with Nm@MSNs-DOX/SM did not significantly change. Moreover, there were no abnormal changes in ALT, AST, BUN, and CRE. Nevertheless, cardiac function index (CK, Myo) and H&E (Fig. 9) indicated that the cardiotoxicity of Nm@MSNs-DOX/SM in nude mice was significantly lower than that of free DOX since the active targeting effect of neutrophil membrane can deliver DOX accumulation in tumor tissue. This is consistent with the previously reported active targeting of neutrophil membrane [70]. However, the histological images of other organs (liver, spleen, lung and kidney) exhibited no abnormalities. To sum up, Nm@MSNs-DOX/SM nanocomposite can improve the hematological toxicity and cardiotoxicity induced by DOX, presenting the potential as a safe and low toxicity anti-tumor nanodrug.

## Discussion

Although the new treatment methods adopted worldwide have improved the prognosis of patients with NHL in varying degrees, most of them are currently expensive, with apparent side effects, high recurrence rate, and unsatisfactory overall treatment effect [71]. Therefore, the search for effective new treatment is still the focus of the current research. Our research provides a new nanodrug delivery system, Nm@MSNs-DOX/SM, which combines chemotherapeutic drugs with anti-inflammatory drugs to target lymphoma. The novel nanocomposite has good biocompatibility and active targeting. The observation of macrophage phagocytosis and drug release efficiency of Nm@MSNs and MSNs indicated that Nm, as a surface modification material, endowed nanocomposites with immune escape ability and excellent tumor active targeting. Consequently, Nm@MSNs-DOX/SM with a long cycle time in vivo can not only reduce the removal of nanoparticles from the body but also improve the drug release efficiency of nanocomposites, resulting in the accumulation of nano-drugs in tumor tissues. Meanwhile, the nanocomposite adopts mesoporous silica as a carrier to make it have high drug loading.

Nm@MSNs-DOX/SM is actively targeted to the tumor site through the membrane and internalized by tumor cells. Based on the weakly acidic characteristics of tumor tissue [72], Nm@MSNS-DOX/SM releases DOX and SM slowly in normal physiological environment, beneficial to prolong the time of blood circulation and increase the enrichment in the tumor site. However, it can quickly release DOX and SM in the weakly acidic environment of the tumor once it reaches the tumor tissue, exerting anti-tumor and anti-inflammatory activities. The nanodrug can realize the controllable release of DOX and SM with the help of the pH gradient between the systemic circulation and the tumor microenvironment [73]. The relationship between inflammation and tumor is mutual. As early as 1863, some scholars observed the existence of inflammatory cells in tumor tissue and demonstrated that the inflammatory microenvironment caused by pro-inflammatory mediators can promote the occurrence and development of tumor [74]. More and more clinical evidence verifies that systemic inflammatory response can occur after DOX [75], and the levels of inflammatory cytokines such as IL-1β and TNF-α are significantly high, contributing to promoting angiogenesis, inhibiting apoptosis, and inducing DNA damage, resulting in poor prognosis [43]. The anti-tumor results in vivo and in vitro suggested that Nm@MSNs-DOX/SM had a better inhibitory effect on tumor cell proliferation compared to free DOX. This can be explained that Nm@MSNs-DOX/SM can not only exert the function of DOX to kill tumor cells but also regulate the changes of tumor microenvironment by inhibiting the expression of inflammatory factors by SM, and thus enhance the anti-tumor effect.

The level of intracellular ROS is closely related to apoptosis [76]. When cells are stimulated by external drugs, the level of ROS increases significantly and leads to the peroxidation of cell membrane proteins and lipids, causing the decrease in MMP, the increase in cell membrane permeability, and the release of a considerable number of free radicals and cytochrome C, and thus inducing cell apoptosis [77]. Apoptosis can be regulated by mitochondrial-mediated endogenous pathway [78]. Bcl-2 and Bax both belong to the Bcl-2 family; Bcl-2 belongs to anti-apoptotic gene, and Bax belongs to pro-apoptotic gene [79]. Both of them play an essential role in regulating the process of apoptosis through mitochondrial pathway [69]. The results indicated that when Nm@MSNs-DOX/SM was used for anti-lymphoma in vivo and in vitro, the level of ROS in cells increased, the expression of Bcl-2 decreased, and the expression of Bax increased, leading to a decrease in the proportion of Bcl-2/Bax, more Bax gathered in the outer membrane of mitochondria, and a decrease in MMP, and thus promoting the occurrence of apoptosis of lymphoma cells.

In this study, a new type of biomimetic nanocomposite platform, which has good biocompatibility and active targeting and can transfer DOX and SM to the tumor site, was constructed. Besides, it cooperatively induces apoptosis of lymphoma cells by causing the increase in ROS, the decrease in MMP, and the change in tumor inflammatory microenvironment. The mechanism of apoptosis may be achieved by changing the expression of Bcl-2 and Bax protein and reducing the expression of inflammatory cytokines TNF-α and IL-1β. The combination of chemotherapeutic drugs and anti-inflammatory drugs will enhance the efficacy of anti-tumor and enlighten the efficient treatment of tumor. Simultaneously, it can effectively overcome the disadvantage of short half-life of traditional nano-drugs owing to the advantages of biomimetic materials, and reduce the toxicity of chemotherapeutic drugs through its good targeting.

## Conclusion

Nm@MSNs-DOX/SM is a new type of anti-tumor nanodrug, coated with MSNs by Nm and loaded with chemotherapeutic drug (DOX) and anti-inflammatory drug (SM). Nm@MSNs-DOX/SM has many advantages, such as good monodispersity, high drug loading rate, weak acid response, good biological safety, avoiding immune system clearance, and active targeting. Besides, Nm@MSNs-DOX/SM kills tumor cells through the Bcl-2/Bax/ROS. The nano-drug can also inhibit the expression of TNF-α/IL-1β and reshape the tumor cell TME to enhance the anti-tumor effect and reduce the toxic and side effects caused by drug alone. Because of these characteristics, Nm@MSNs-DOX/SM is a safe and efficient targeted drug delivery system, which may be used in the effective treatment of lymphoma.

## Supplementary Information

**Additional file 1.** Additional figures S1–S11.

## Data Availability

All data generated or analyzed during this study are included in this published article.

## References

[1] Liu E, Marin D, Banerjee P, Macapinlac HA, Thompson P, Basar R, Nassif Kerbauy L, Overman B, Thall P, Kaplan M (2020). Use of CAR-transduced natural killer cells in CD19-positive lymphoid tumors. N Engl J Med.

[2] Moleti ML, Testi AM, Foa R (2020). Treatment of relapsed/refractory paediatric aggressive B-cell non-Hodgkin lymphoma. Br J Haematol.

[3] Jardin F (2019). Improving R-CHOP in diffuse large B-cell lymphoma is still a challenge. Lancet Oncol.

[4] Carvalho C, Santos RX, Cardoso S, Correia S, Oliveira PJ, Santos MS, Moreira PI (2009). Doxorubicin: the good, the bad and the ugly effect. Curr Med Chem.

[5] Toy R, Bauer L, Hoimes C, Ghaghada KB, Karathanasis E (2014). Targeted nanotechnology for cancer imaging. Adv Drug Deliv Rev.

[6] Karthik S, Puvvada N, Kumar BN, Rajput S, Pathak A, Mandal M, Singh ND (2013). Photoresponsive coumarin-tethered multifunctional magnetic nanoparticles for release of anticancer drug. ACS Appl Mater Interfaces.

[7] Zhang Y, Ma S, Liu X, Xu Y, Zhao J, Si X, Li H, Huang Z, Wang Z, Tang Z (2021). Supramolecular assembled programmable nanomedicine as in situ cancer vaccine for cancer immunotherapy. Adv Mater.

[8] Zhao Q, Sun XY, Wu B, Shang Y, Huang X, Dong H, Liu H, Chen W, Gui R, Li J (2021). Construction of biomimetic silver nanoparticles in the treatment of lymphoma. Mater Sci Eng C.

[9] Garcia-Bennett AE (2011). Synthesis, toxicology and potential of ordered mesoporous materials in nanomedicine. Nanomedicine.

[10] Hassankhani R, Esmaeillou M, Tehrani AA, Nasirzadeh K, Khadir F, Maadi H (2015). In vivo toxicity of orally administrated silicon dioxide nanoparticles in healthy adult mice. Environ Sci Pollut Res Int.

[11] Zhao Q, Sun X, Wu B, Shang Y, Huang X, Dong H, Liu H, Chen W, Gui R, Li J (2021). Construction of homologous cancer cell membrane camouflage in a nano-drug delivery system for the treatment of lymphoma. J Nanobiotechnol.

[12] Tang F, Li L, Chen D (2012). Mesoporous silica nanoparticles: synthesis, biocompatibility and drug delivery. Adv Mater.

[13] Slowing II, Vivero-Escoto JL, Wu CW, Lin VS (2008). Mesoporous silica nanoparticles as controlled release drug delivery and gene transfection carriers. Adv Drug Deliv Rev.

[14] Mekaru H, Lu J, Tamanoi F (2015). Development of mesoporous silica-based nanoparticles with controlled release capability for cancer therapy. Adv Drug Deliv Rev.

[15] Liu X, Situ A, Kang Y, Villabroza KR, Liao Y, Chang CH, Donahue T, Nel AE, Meng H (2016). Irinotecan delivery by lipid-coated mesoporous silica nanoparticles shows improved efficacy and safety over liposomes for pancreatic cancer. ACS Nano.

[16] Hanahan D, Weinberg RA (2011). Hallmarks of cancer: the next generation. Cell.

[17] Karki R, Kanneganti TD (2019). Diverging inflammasome signals in tumorigenesis and potential targeting. Nat Rev Cancer.

[18] Marelli G, Sica A, Vannucci L, Allavena P (2017). Inflammation as target in cancer therapy. Curr Opin Pharmacol.

[19] Yang L, Lin PC (2017). Mechanisms that drive inflammatory tumor microenvironment, tumor heterogeneity, and metastatic progression. Semin Cancer Biol.

[20] Sharma P, Allison JP (2015). Immune checkpoint targeting in cancer therapy: toward combination strategies with curative potential. Cell.

[21] Ghiringhelli F, Apetoh L, Tesniere A, Aymeric L, Ma Y, Ortiz C, Vermaelen K, Panaretakis T, Mignot G, Ullrich E (2009). Activation of the NLRP3 inflammasome in dendritic cells induces IL-1beta-dependent adaptive immunity against tumors. Nat Med.

[22] Zhang Z, Lin G, Yan Y, Li X, Hu Y, Wang J, Yin B, Wu Y, Li Z, Yang XP (2018). Transmembrane TNF-alpha promotes chemoresistance in breast cancer cells. Oncogene.

[23] Zhu B, Gong N, Fan H, Peng CS, Ding XJ, Jiang Y, Wang YX (2014). Lamiophlomis rotata, an orally available Tibetan herbal painkiller, specifically reduces pain hypersensitivity states through the activation of spinal glucagon-like peptide-1 receptors. Anesthesiology.

[24] Zhang L, Kan ZC, Zhang XL, Fang H, Jiang WL (2014). 8-O-acetyl shanzhiside methylester attenuates cerebral ischaemia/reperfusion injury through an anti-inflammatory mechanism in diabetic rats. Basic Clin Pharmacol Toxicol.

[25] Sun T, Luo L, Tian QQ, Wang WJ, Yang Q (2020). Anxiolytic effects of 8-*O*-acetyl shanzhiside methylester on acute and chronic anxiety via inflammatory response inhibition and excitatory/inhibitory transmission imbalance. Neurotox Res.

[26] Kang ZC, Jiang WL, Xu Y, Zhu HB, Hou J (2012). Cardioprotection with 8-*O*-acetyl shanzhiside methylester on experimental myocardial ischemia injury. Eur J Pharm Sci.

[27] Wojnilowicz M, Glab A, Bertucci A, Caruso F, Cavalieri F (2019). Super-resolution imaging of proton sponge-triggered rupture of endosomes and cytosolic release of small interfering RNA. ACS Nano.

[28] Hu Q, Sun W, Qian C, Wang C, Bomba HN, Gu Z (2015). Anticancer platelet-mimicking nanovehicles. Adv Mater.

[29] Hu Q, Qian C, Sun W, Wang J, Chen Z, Bomba HN, Xin H, Shen Q, Gu Z (2016). Engineered nanoplatelets for enhanced treatment of multiple myeloma and thrombus. Adv Mater.

[30] Yu B, Goel S, Ni D, Ellison PA, Siamof CM, Jiang D, Cheng L, Kang L, Yu F, Liu Z (2018). Reassembly of (89) Zr-labeled cancer cell membranes into multicompartment membrane-derived liposomes for PET-trackable tumor-targeted theranostics. Adv Mater.

[31] Pop V, Seicean A, Soritau O, Buiga R, Barsan M, Balacescu L, Burz C (2019). Interleukin-6 correlated with neutrophil-to-lymphocyte ratio in pancreatic cancer. Ann Oncol.

[32] Giese MA, Hind LE, Huttenlocher A (2019). Neutrophil plasticity in the tumor microenvironment. Blood.

[33] Zhang Q, Dehaini D, Zhang Y, Zhou J, Chen X, Zhang L, Fang RH, Gao W, Zhang L (2018). Neutrophil membrane-coated nanoparticles inhibit synovial inflammation and alleviate joint damage in inflammatory arthritis. Nat Nanotechnol.

[34] Kang T, Zhu Q, Wei D, Feng J, Yao J, Jiang T, Song Q, Wei X, Chen H, Gao X, Chen J (2017). Nanoparticles coated with neutrophil membranes can effectively treat cancer metastasis. ACS Nano.

[35] Zhao Q, Li J, Wu B, Shang Y, Huang X, Dong H, Liu H, Chen W, Gui R, Nie X (2020). Smart biomimetic nanocomposites mediate mitochondrial outcome through aerobic glycolysis reprogramming: a promising treatment for lymphoma. ACS Appl Mater Interfaces.

[36] Cao H, Dan Z, He X, Zhang Z, Yu H, Yin Q, Li Y (2016). Liposomes coated with isolated macrophage membrane can target lung metastasis of breast cancer. ACS Nano.

[37] Chen RX, Chen X, Xia LP, Zhang JX, Pan ZZ, Ma XD, Han K, Chen JW, Judde JG, Deas O (2019). N(6)-methyladenosine modification of circNSUN2 facilitates cytoplasmic export and stabilizes HMGA2 to promote colorectal liver metastasis. Nat Commun.

[38] Yi X, Lian X, Dong J, Wan Z, Xia C, Song X, Fu Y, Gong T, Zhang Z (2015). Co-delivery of pirarubicin and paclitaxel by human serum albumin nanoparticles to enhance antitumor effect and reduce systemic toxicity in breast cancers. Mol Pharm.

[39] Yan F, Li H, Zhong Z, Zhou M, Lin Y, Tang C, Li C (2019). Co-delivery of prednisolone and curcumin in human serum albumin nanoparticles for effective treatment of rheumatoid arthritis. Int J Nanomed.

[40] Li J, Huang X, Huang R, Jiang J, Gui R (2019). Erythrocyte membrane camouflaged graphene oxide for tumor-targeted photothermal-chemotherapy. Carbon.

[41] Moreno-Villaecija MA, Sedo-Vegara J, Guisasola E, Baeza A, Regi MV, Nador F, Ruiz-Molina D (2018). Polydopamine-like coatings as payload gatekeepers for mesoporous silica nanoparticles. ACS Appl Mater Interfaces.

[42] Rabadi SM, Sanchez BC, Varanat M, Ma Z, Catlett SV, Melendez JA, Malik M, Bakshi CS (2016). Antioxidant defenses of francisella tularensis modulate macrophage function and production of proinflammatory cytokines. J Biol Chem.

[43] Huang X, Wu B, Li J, Shang Y, Chen W, Nie X, Gui R (2019). Anti-tumour effects of red blood cell membrane-camouflaged black phosphorous quantum dots combined with chemotherapy and anti-inflammatory therapy. Artif Cells Nanomed Biotechnol.

[44] Zhao Q, Li J, Wu B, Shang Y, Huang X, Dong H, Liu H, Gui R, Nie X (2020). A nano-traditional chinese medicine against lymphoma that regulates the level of reactive oxygen species. Front Chem.

[45] Song G, Zheng X, Wang Y, Xia X, Chu S, Rao J (2019). A magneto-optical nanoplatform for multimodality imaging of tumors in mice. ACS Nano.

[46] Kang L, Li C, Rosenkrans ZT, Huo N, Chen Z, Ehlerding EB, Huo Y, Ferreira CA, Barnhart TE, Engle JW (2021). CD38-targeted theranostics of lymphoma with 89Zr/177Lu-labeled daratumumab. Adv Sci.

[47] Ottewell PD, Monkkonen H, Jones M, Lefley DV, Coleman RE, Holen I (2008). Antitumor effects of doxorubicin followed by zoledronic acid in a mouse model of breast cancer. J Natl Cancer Inst.

[48] Cai D, Liu L, Han C, Ma X, Qian J, Zhou J, Zhu W (2019). Cancer cell membrane-coated mesoporous silica loaded with superparamagnetic ferroferric oxide and Paclitaxel for the combination of Chemo/Magnetocaloric therapy on MDA-MB-231 cells. Sci Rep.

[49] Xuan M, Shao J, Dai L, He Q, Li J (2015). Macrophage cell membrane camouflaged mesoporous silica nanocapsules for in vivo cancer therapy. Adv Healthc Mater.

[50] Akita H, Kudo A, Minoura A, Yamaguti M, Khalil IA, Moriguchi R, Masuda T, Danev R, Nagayama K, Kogure K, Harashima H (2009). Multi-layered nanoparticles for penetrating the endosome and nuclear membrane via a step-wise membrane fusion process. Biomaterials.

[51] Chen B, Dai W, He B, Zhang H, Wang X, Wang Y, Zhang Q (2017). Current multistage drug delivery systems based on the tumor microenvironment. Theranostics.

[52] Tekade RK, Sun X (2017). The Warburg effect and glucose-derived cancer theranostics. Drug Discov Today.

[53] Coussens LM, Werb Z (2002). Inflammation and cancer. Nature.

[54] Logtenberg MEW, Scheeren FA, Schumacher TN (2020). The CD47-SIRPalpha immune checkpoint. Immunity.

[55] Chao MP, Alizadeh AA, Tang C, Myklebust JH, Varghese B, Gill S, Jan M, Cha AC, Chan CK, Tan BT (2010). Anti-CD47 antibody synergizes with rituximab to promote phagocytosis and eradicate non-Hodgkin lymphoma. Cell.

[56] Matlung HL, Szilagyi K, Barclay NA, van den Berg TK (2017). The CD47-SIRPalpha signaling axis as an innate immune checkpoint in cancer. Immunol Rev.

[57] Brown EJ, Frazier WA (2001). Integrin-associated protein (CD47) and its ligands. Trends Cell Biol.

[58] Kimura S, Tada N, Liu-Lam Y, Hammerling U (1984). Studies of the mouse Ly-6 alloantigen system. II. Complexities of the Ly-6 region. Immunogenetics.

[59] Sunderkotter C, Nikolic T, Dillon MJ, Van Rooijen N, Stehling M, Drevets DA, Leenen PJ (2004). Subpopulations of mouse blood monocytes differ in maturation stage and inflammatory response. J Immunol.

[60] Loughner CL, Bruford EA, McAndrews MS, Delp EE, Swamynathan S, Swamynathan SK (2016). Organization, evolution and functions of the human and mouse Ly6/uPAR family genes. Hum Genomics.

[61] Serbina NV, Pamer EG (2006). Monocyte emigration from bone marrow during bacterial infection requires signals mediated by chemokine receptor CCR2. Nat Immunol.

[62] Fumagalli L, Zhang H, Baruzzi A, Lowell CA, Berton G (2007). The Src family kinases Hck and Fgr regulate neutrophil responses to N-formyl-methionyl-leucyl-phenylalanine. J Immunol.

[63] Hatakeyama S, Iwabuchi K, Ogasawara K, Good RA, Onoe K (1994). The murine c-fgr gene product associated with Ly6C and p70 integral membrane protein is expressed in cells of a monocyte/macrophage lineage. Proc Natl Acad Sci USA.

[64] Chou TC, Talalay P (1984). Quantitative analysis of dose-effect relationships: the combined effects of multiple drugs or enzyme inhibitors. Adv Enzyme Regul.

[65] Traverso N, Ricciarelli R, Nitti M, Marengo B, Furfaro AL, Pronzato MA, Marinari UM, Domenicotti C (2013). Role of glutathione in cancer progression and chemoresistance. Oxid Med Cell Longev.

[66] Pavithra PS, Mehta A, Verma RS (2018). Induction of apoptosis by essential oil from *P. missionis* in skin epidermoid cancer cells. Phytomedicine.

[67] Liu Y, Zhang X, Zhou M, Nan X, Chen X, Zhang X (2017). Mitochondrial-targeting lonidamine-doxorubicin nanoparticles for synergistic chemotherapy to conquer drug resistance. ACS Appl Mater Interfaces.

[68] Nguyen HV, Vandenberg CJ, Ng AP, Robati MR, Anstee NS, Rimes J, Hawkins ED, Cory S (2020). Development and survival of MYC-driven lymphomas require the MYC antagonist MNT to curb MYC-induced apoptosis. Blood.

[69] Popgeorgiev N, Sa JD, Jabbour L, Banjara S, Nguyen TTM, Akhavan ESA, Gadet R, Ralchev N, Manon S, Hinds MG (2020). Ancient and conserved functional interplay between Bcl-2 family proteins in the mitochondrial pathway of apoptosis. Sci Adv.

[70] Chu D, Dong X, Shi X, Zhang C, Wang Z (2018). Neutrophil-based drug delivery systems. Adv Mater.

[71] Camicia R, Winkler HC, Hassa PO (2015). Novel drug targets for personalized precision medicine in relapsed/refractory diffuse large B-cell lymphoma: a comprehensive review. Mol Cancer.

[72] Liu J, Wu Y, Fu C, Li B, Li L, Zhang R, Xu T, Xu ZP (2020). Charge reversion simultaneously enhances tumor accumulation and cell uptake of layered double hydroxide nanohybrids for effective imaging and therapy. Small.

[73] Hou L, Tian C, Yan Y, Zhang L, Zhang H, Zhang Z (2020). Manganese-based nanoactivator optimizes cancer immunotherapy via enhancing innate immunity. ACS Nano.

[74] Klichinsky M, Ruella M, Shestova O, Lu XM, Best A, Zeeman M, Schmierer M, Gabrusiewicz K, Anderson NR, Petty NE (2020). Human chimeric antigen receptor macrophages for cancer immunotherapy. Nat Biotechnol.

[75] Qi W, Boliang W, Xiaoxi T, Guoqiang F, Jianbo X, Gang W (2020). Cardamonin protects against doxorubicin-induced cardiotoxicity in mice by restraining oxidative stress and inflammation associated with Nrf2 signaling. Biomed Pharmacother.

[76] Hayes JD, Dinkova-Kostova AT, Tew KD (2020). Oxidative stress in cancer. Cancer Cell.

[77] Zhang J, Yang J, Zuo T, Ma S, Xokrat N, Hu Z, Wang Z, Xu R, Wei Y, Shen Q (2021). Heparanase-driven sequential released nanoparticles for ferroptosis and tumor microenvironment modulations synergism in breast cancer therapy. Biomaterials.

[78] Fresquet V, Garcia-Barchino MJ, Larrayoz MJ, Celay J, Vicente C, Fernandez-Galilea M, Larrayoz MJ, Calasanz MJ, Panizo C, Junza A (2020). Endogenous retroelement activation by epigenetic therapy reverses the Warburg effect and elicits mitochondrial-mediated cancer cell death. Cancer Discov.

[79] Li X, Wang J, Gong X, Zhang M, Kang S, Shu B, Wei Z, Huang ZS, Li D (2020). Upregulation of BCL-2 by acridone derivative through gene promoter i-motif for alleviating liver damage of NAFLD/NASH. Nucleic Acids Res.

