# Supplementary material for: Biomimetic nanotherapy: core–shell structured nanocomplexes based on the neutrophil membrane for targeted therapy of lymphoma
Source: J Nanobiotechnology. 2021 Jun 13;19:179. doi: 10.1186/s12951-021-00922-4 (PMC8201715; doi:10.1186/s12951-021-00922-4)
Supplement: Supplementary file 1 — Additional file 1. Additional figures S1–S11. [file 12951_2021_922_MOESM1_ESM.docx]

**Additional file 1**

**Biomimetic nanotherapy:** **core-shell structured nanocomplexes based on the neutrophil membrane for targeted therapy of lymphoma**

Qiangqiang Zhao^a,b^, Duanfeng Jiang^c^, Xiaoying sun^d,e^, Qiuyu Mo^f^, Shaobin Chen^b^, Wansong Chen^g^ , Rong Gui^a^^[[1]](#footnote-1)*^and Xianjun Ma^h^^[[2]](#footnote-2)^*

^a^ Department of Blood Transfusion, the Third Xiangya Hospital, Central South University, Changsha 410013, P. R. China

^b^ Department of Hematology, the Qinghai Provincial People's Hospital, Xining

810007, P. R. China

^c^ Department of Hematology, the Third Xiangya Hospital, Central South University, Changsha 410013, P. R. China

^d^ Nursing School, Soochow University, Suzhou 215000, P. R. China

^e^ Department of Emergency, the Qinghai Provincial People's Hospital, Xining

810007, P. R. China

^f^ Department of Hematology, Affiliated Hospital of Guilin Medical University, Guilin 541002, P. R. China

^g^ College of Chemistry and Chemical Engineering, Central South University, Changsha 410083, P. R. China

^h^ Department of Blood Transfusion, Qilu Hospital of Shandong University, Jinan 250012, P. R. China.

1. **Additional Results**


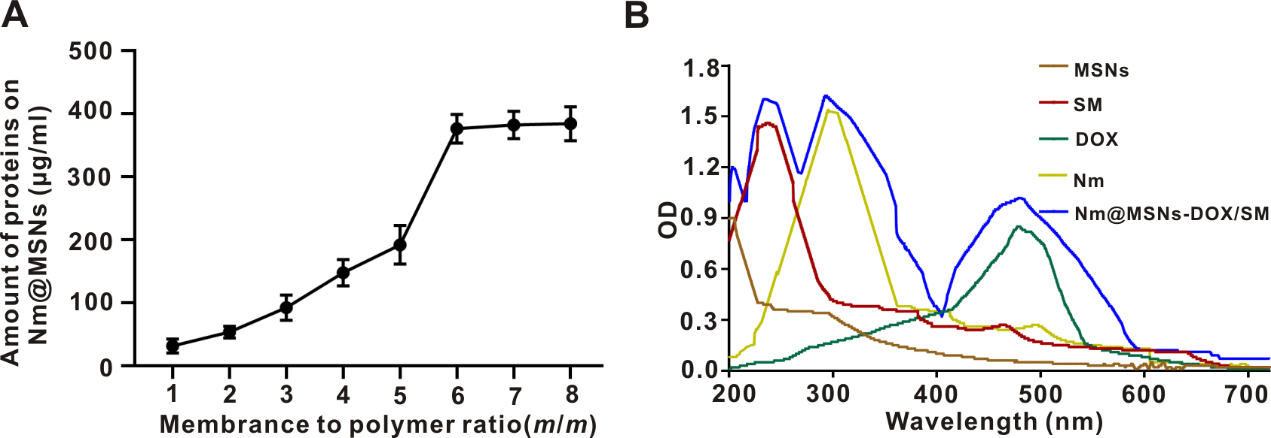


**Figure S1.** (A) Determination of total membrane protein content on Nm@MSNs. (B) UV-Vis spectra of MSNs, SM, DOX, Neutrophil membrane (Nm) vesicles and Nm@MSNs-DOX/SM.


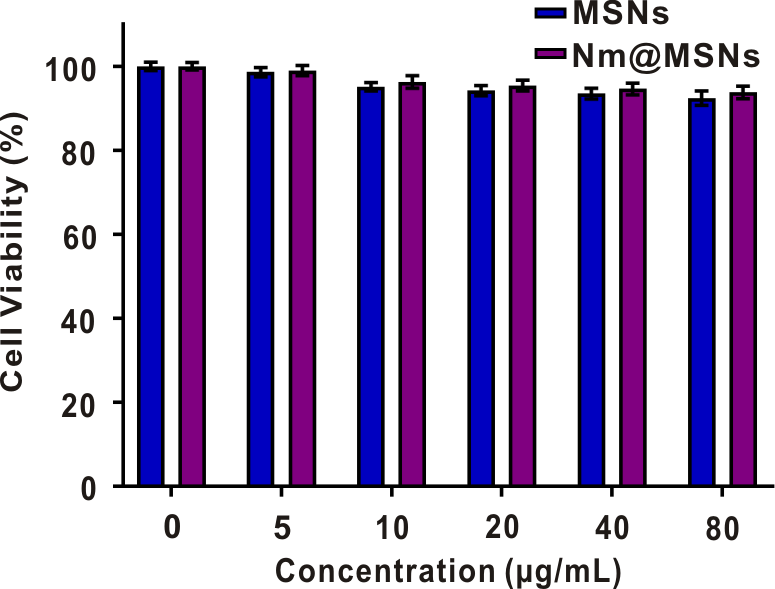


**Figure S2.** The cell viability of SU-DHL-2 cells treated with different concentrations of MSNs and Nm@MSNs. Data are mean ± SD (n=3).


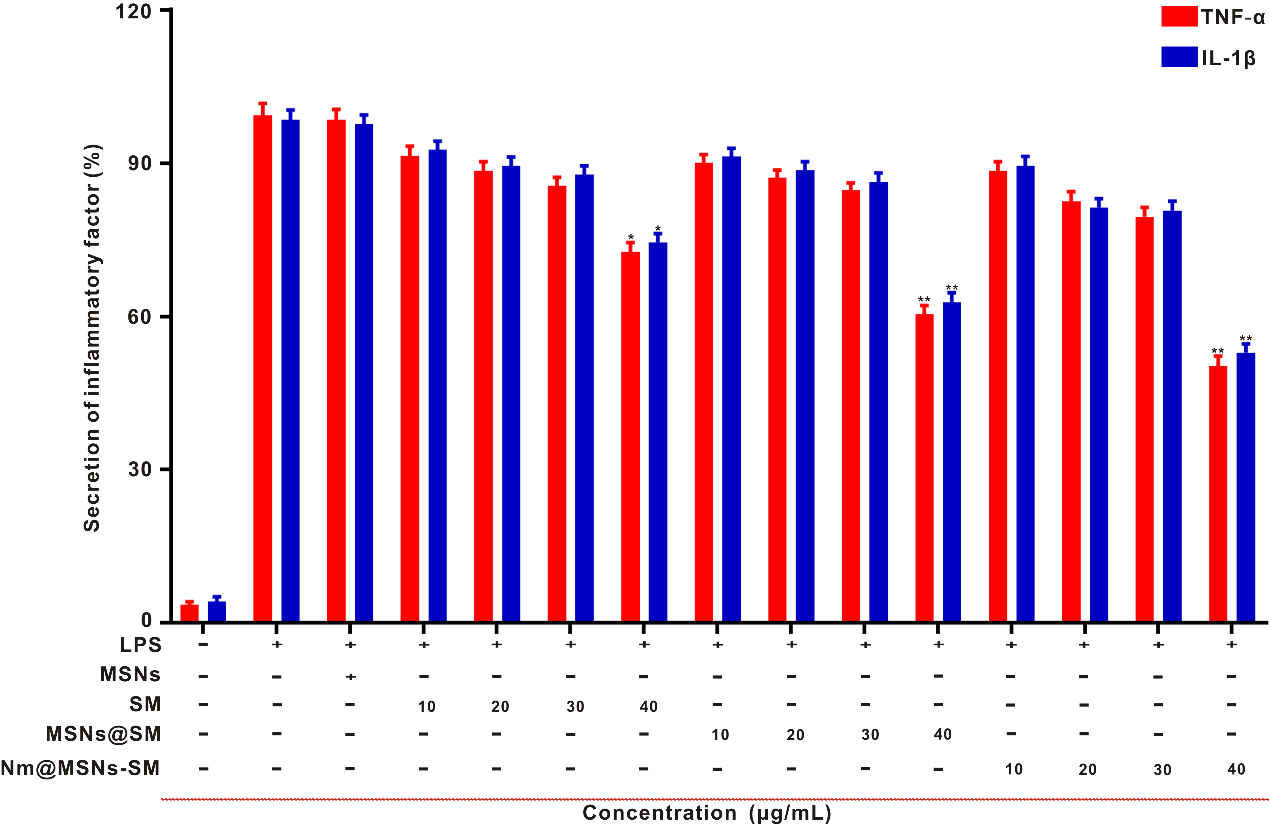


**Figure S3.**TNF-α and IL-1β secretion at different treatment. The TNF-α an IL-1β secretion of macrophages treated by PBS (negative control) and lipopolysaccharides (LPS) (positive control) was set as 0% and 100%, respectively.Data are mean ± SD (n=3). (* *p*<0.05, ** *p*<0.01).


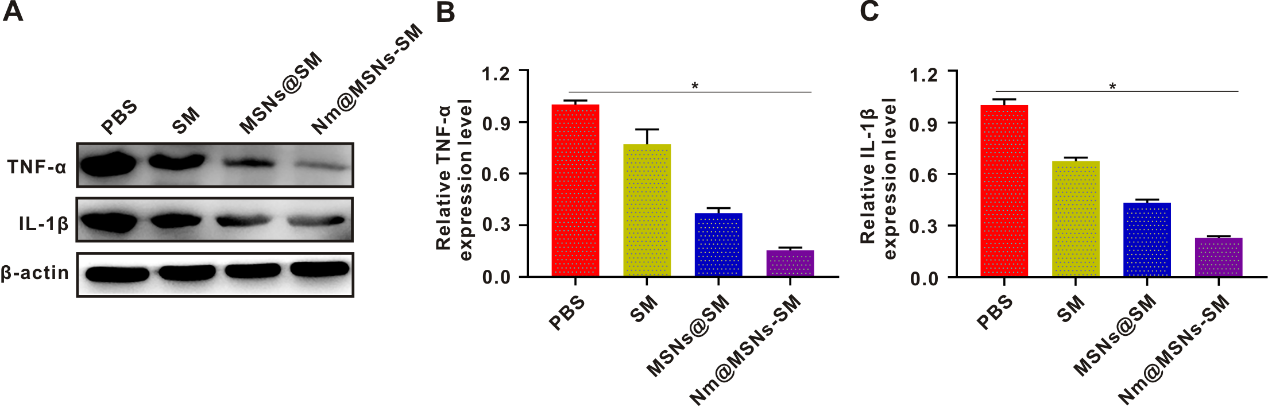


**Figure S4.** (A) The expression of TNF-α and IL-1β were detected by Western Blot. (B-C) Semi-quantitative analysis of Western Blot bands by Image J software. Data are presented as means ± SD (n=3). (* *p*<0.05).


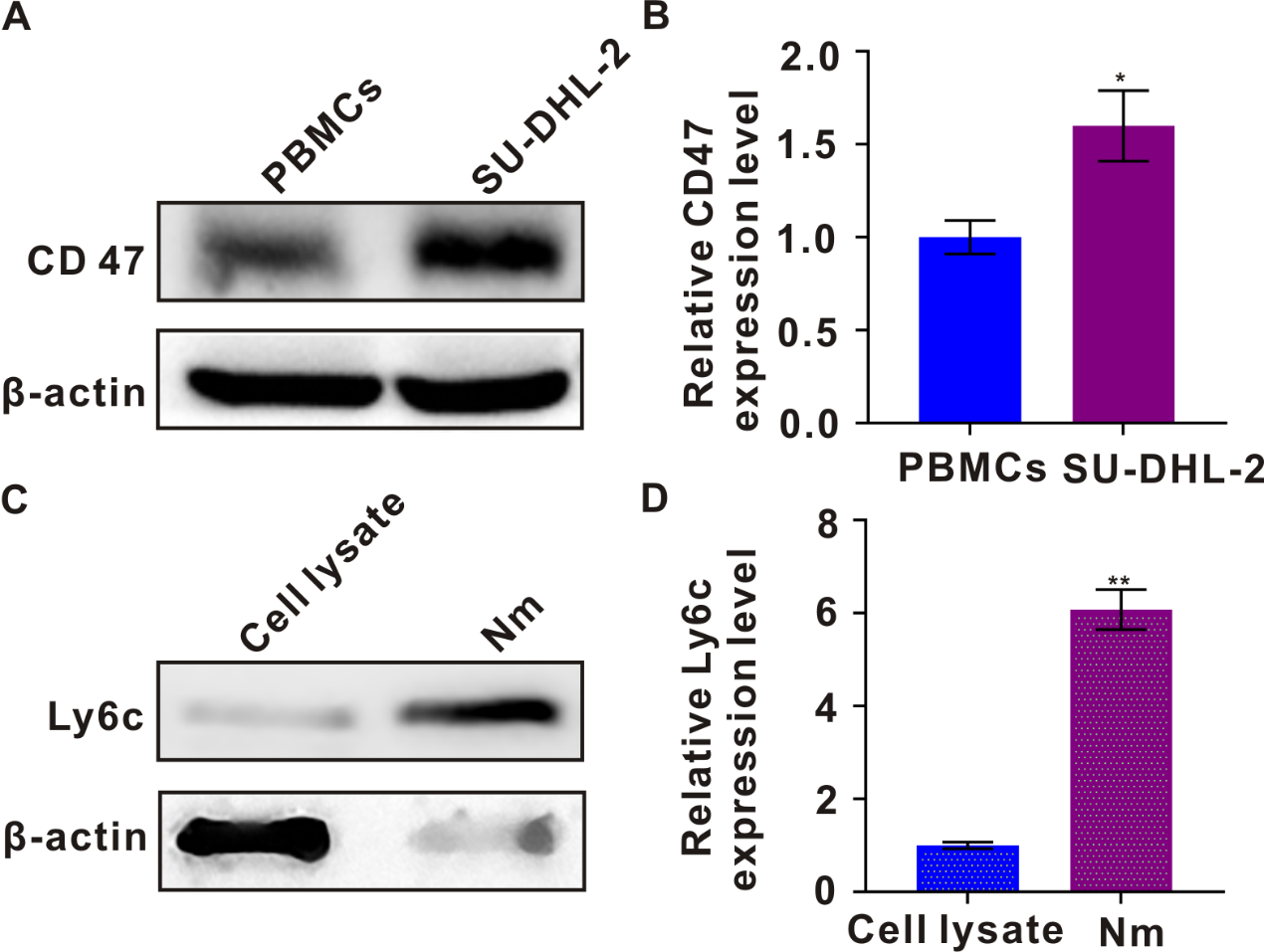


**Figure S5.** (A) The expression of CD47 in PBMCs and SU-DHL-2 cells were detected by Western Blot. (B) Semi-quantitative analysis of Western Blot bands by Image J software. (C) Detection of neutrophil membrane specific Ly6c by the Western blotting assay. (B) Semi-quantitative analysis of Western Blot bands by Image J software. Data are presented as means ± SD (n=3). (* *p*<0.05, ** *p*<0.01).


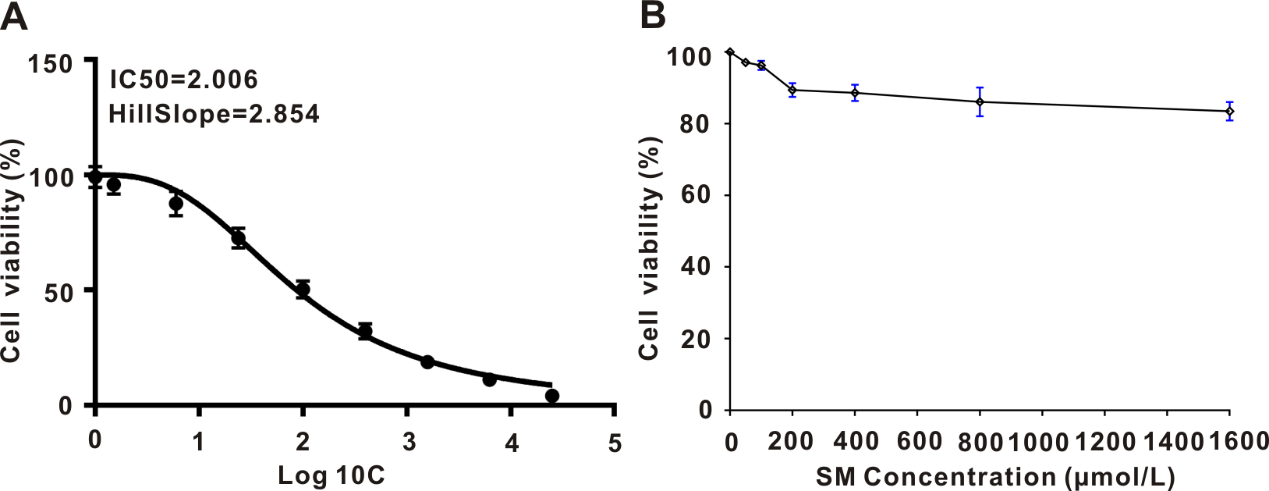


**Figure S6.** (A) Cell viability of different concentrations of DOX on SU-HDL-2 cells. (B) Cell viability of SU-DHL-2 cells treated with different concentrations of SM. Data are mean ± SD (n=3).


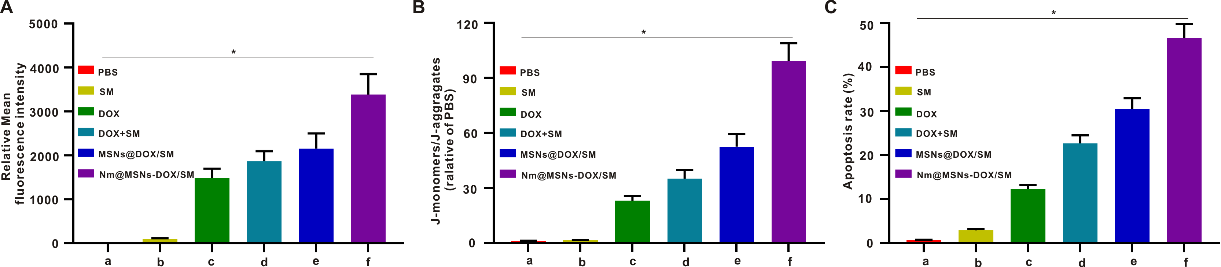


**Figure S7.** SU-DHL-2 cells were treated with a) PBS, b) SM, c) DOX, d) DOX+SM, e) MSNs@DOX/SM and f) Nm@MSNs-DOX/SM for 24 h, respectively. (A) Relative mean fluorescene intensity of ROS. (B) JC-monomers/JC-aggragates (relative of PBS). (C) Percentage of apoptotic. Data are presented as the mean ± SD (n = 3). (* *p*<0.05).


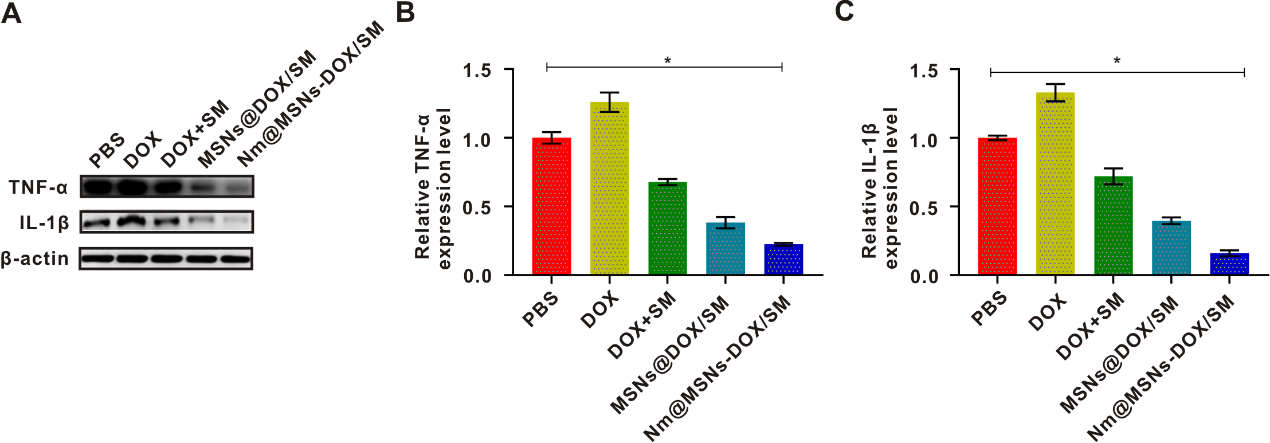


**Figure S8 (A-C).** The expression of apoptosis-related proteins TNF-α and IL-1β were detected by Western blotting. Data are presented as the mean ± SD (n=3). (**p*<0.05).


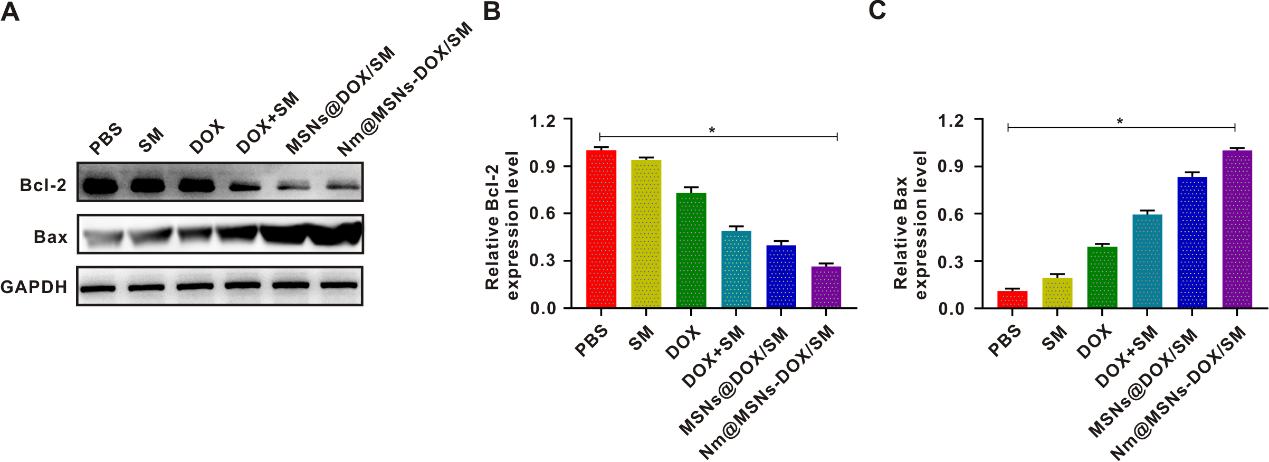


**Figure S9 (A-C).** The expression of apoptosis-related proteins Bcl-2 and Bax were detected by Western blotting. Data are presented as the mean ± SD (n=3). (**p*<0.05).


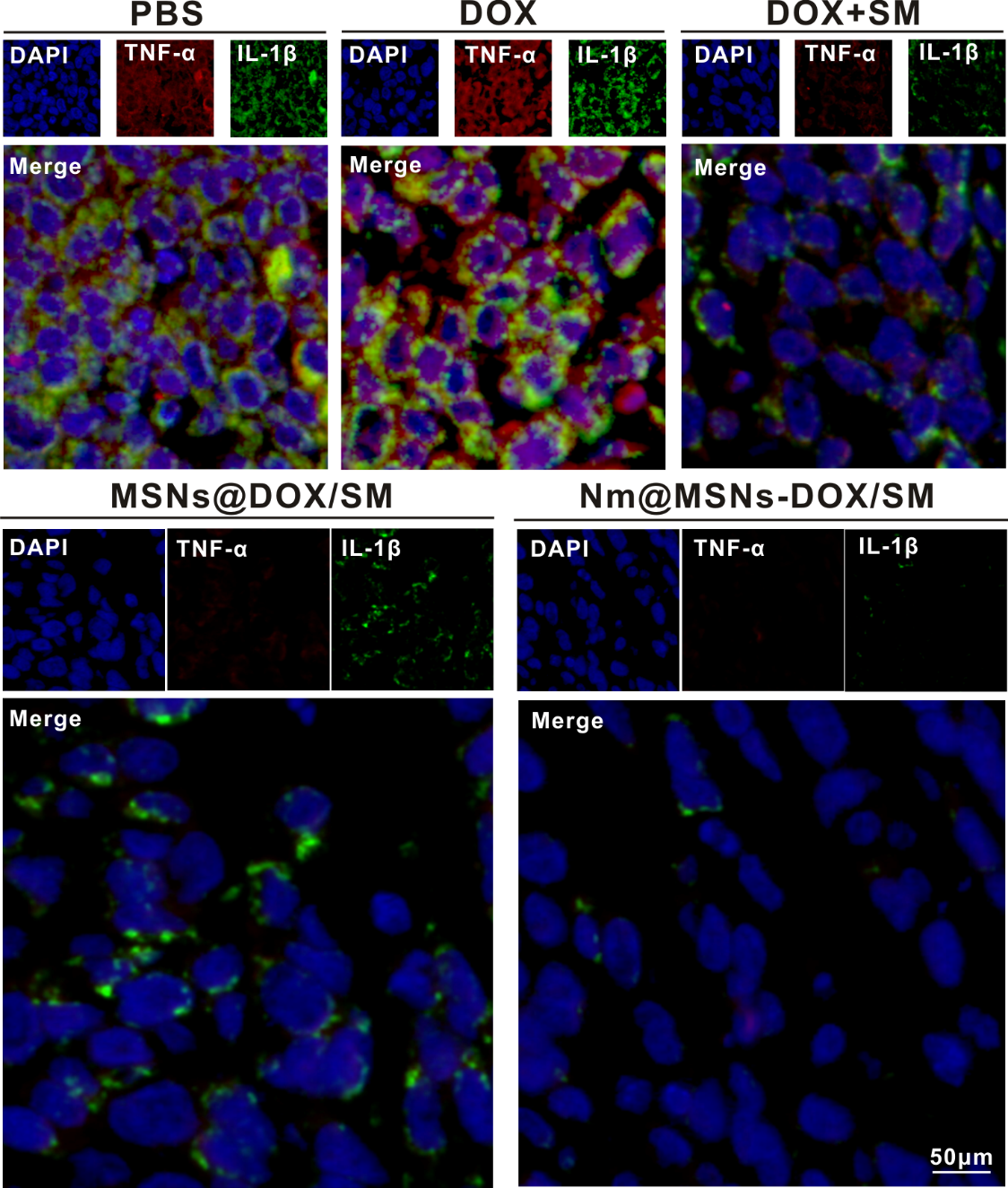


**Figure S10.** Anti-inflammatory effect of Nm@MSNs-DOX/SM.The LCFM images of the tumor area were taken after different treatments.Red:TNF-a, Green: IL-1β, Blue: Nucleus. Scale bar: 50 µm.


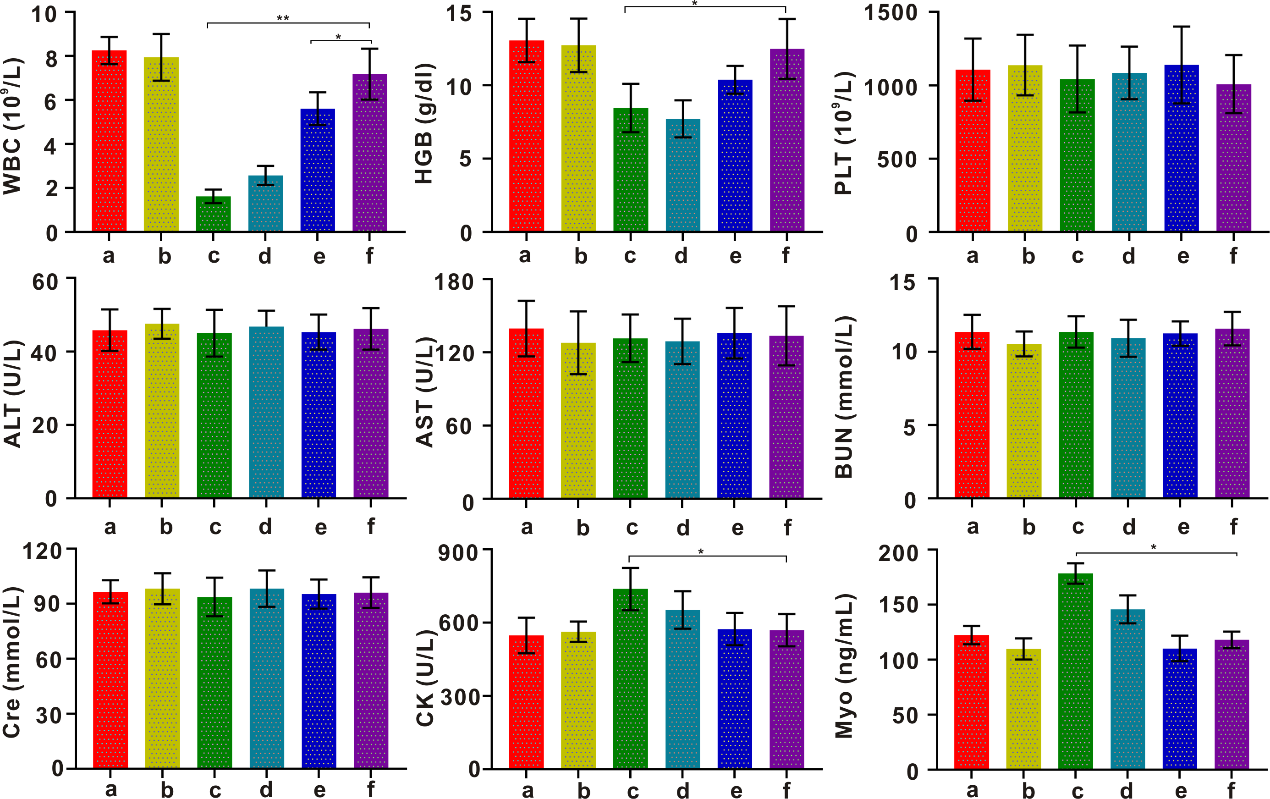


**Figure S11.** The nude mice were euthanized on the 15st day after a) PBS, b) SM, c) DOX, d) DOX+SM, e) MSNs@DOX/SM and f) Nm@MSNs-DOX/SM, respectively, were injected into the tail vein, and the blood routine indexes (WBC, HGB, and PLT) and blood biochemical indexes (ALT,AST, BUN, CRE, CK and Myo) were detected. Data are presented as mean ± SD (n = 3). (* *p*<0.05, ** *p*<0.01).

1. * Corresponding author. Phone/Fax: +86-731-8861 8513.

   E-mail address: zndxgr@163.com (R. Gui) [↑](#footnote-ref-1)
2. * Corresponding author. Phone/Fax: +86-18560087567

   E-mail address: zgjyws@126.com (X. J. Ma) [↑](#footnote-ref-2)
